# Dynamics of the gut-liver axis in rats with varying fibrosis severity

**DOI:** 10.7150/ijbs.69833

**Published:** 2022-05-09

**Authors:** Hongyan Xiang, Zongyi Liu, Huanyu Xiang, Dejuan Xiang, Shuang Xiao, Jing Xiao, Wei Shen, Peng Hu, Hong Ren, Mingli Peng

**Affiliations:** Key Laboratory of Molecular Biology for Infectious Diseases (Ministry of Education), Institute for Viral Hepatitis, Department of Infectious Diseases, The Second Affiliated Hospital, Chongqing Medical University, Chongqing 400010, China.

**Keywords:** gut-liver axis, liver fibrosis, gut microbiota, bile acid, gut barrier

## Abstract

The classic carbon tetrachloride (CCl_4_)-induced liver injury model is widely used to study the pathogenesis of fibrosis and evaluate anti-fibrosis drugs. Here, we investigated the dynamic changes in the gut microbiota, bile acids (BAs) and the gut barrier over different fibrosis severities in a CCl_4_-based model. 16S rDNA sequencing demonstrated that the beneficial taxon Lactobacillus was always underrepresented, and pathogens including *Escherichia_Shigella*, *Clostridium_sensu_stricto_1*, *Colidextribacter*, and *Lachnospiraceae_UCG_010* were significantly overrepresented across liver fibrosis severities. Gut dysbiosis was more severe at the early stage of liver injury and advanced stage of fibrosis. An ultra-performance liquid chromatography-tandem mass spectrometry (UPLC-MS/MS) analysis revealed that with the progress of fibrosis, unconjugated BAs in faeces were significantly decreased and conjugated BAs in serum were significantly increased. The FXR-SHP signalling pathway in the liver and ileum was statistically repressed in the fibrosis groups. Determination of lipopolysaccharide (LPS) and fluorescein isothiocyanate (FITC)-dextran levels in plasma showed that the intestinal barrier remained relatively intact in the advanced fibrosis stage. The advances in knowledge of the gut-liver axis provided by this study yield new insights for application in research and drug evaluation.

## Introduction

The gut-liver axis refers to the anatomical and functional relationship between the liver and the intestine. The gut microbiota, microbial metabolites and bile acids (BAs) are the main signals in the axis, while the gut mucosal and vascular barriers provide an environment for the interactions between the gut and the liver 1. The gut-liver axis has been demonstrated to be involved in the pathogenesis of prevalent liver diseases, such as alcohol-associated liver disease (ALD), nonalcoholic fatty liver disease (NAFLD), cholestatic liver diseases, and hepatocellular carcinoma (HCC), affecting the degree of hepatic inflammation, steatosis, fibrosis/cirrhosis and complications of cirrhosis 2, 3.

Disruption of the gut-liver axis is the pathophysiological basis for many chronic liver diseases (CLDs). The main features of a disrupted gut-liver axis include an altered intestinal microbiota, changes in luminal levels of BAs, and gut barrier damage 4. Dysregulation of the gut microbiota is the cornerstone of gut-liver axis disruption in CLDs, which could result in subsequent alterations in the levels of gut metabolites (secondary BAs, ethanol, and trimethylamine) and intestinal barrier dysfunction 4, 5. Gut dysbiosis-related metabolism, especially BA alteration, contributes to alcohol-associated injury 6. Alterations in microbiota composition and bacterial overgrowth challenge a hyperpermeable intestinal barrier with an overload of pathogen-associated molecular patterns (PAMPs) or metabolites such as trimethylamine in NAFLD, which elicit liver inflammation 1, 7. Patients with cirrhosis have revealed reduced bacterial diversity and a shift in family structure towards greater abundances of potentially pathogenic taxa such as *Enterococcaceae* and *Staphylococcaceae* and lower abundances of beneficial autochthonous taxa such as* Lachnospiraceae* and *Ruminococcaceae* 1. Intestinal BA flow is reduced in patients with cirrhosis, which in turn aggravates the intestinal flora disorder and changes in BA composition, ultimately suppressing intestinal FXR signalling, which compromises mucus and antimicrobial peptide synthesis and gut-vascular barrier integrity 8, 9.

Due to the gradual clarification of the crosstalk between the gut-liver axis and liver disease, interventions targeting components of the gut-liver axis such as intestinal content, the intestinal microbiome and the intestinal mucosa, to treat chronic liver injury are the future trends 10, 11. Currently, the classic animal model for evaluating anti-fibrotic drugs is the carbon tetrachloride (CCl_4_)-induced liver injury model, which has characteristics similar to those of the human liver fibrosis process caused by toxic damage 12, 13. However, the changes in the gut-liver axis in this model itself across a range of fibrosis severity remain unclear. Here, this study aimed to explore the dynamic changes in the gut-liver axis, including the intestinal microbiota, BAs, and the gut barrier, at different fibrosis stages in CCl_4_-treated rats. The advances in knowledge of the gut-liver axis provided by this study yield new insights for application in research and drug evaluation.

## Materials and methods

### Reagents and antibodies

Antibodies against alpha-smooth muscle actin (α-SMA, ab124964), collagen I (COL1A1, ab260043), cytochrome P450 family 8 subfamily B member 1 (CYP8B1, ab191910), and goat anti-rabbit IgG (ab6712) were purchased from Abcam Inc. (Cambridge, UK); Antibodies to cytochrome P450 family 7 subfamily A member 1 (CYP7A1, A10615), farnesoid X receptor (FXR, A12788*)*, small heterodimer partner (SHP, A1836), glyceraldehyde-3-phosphate dehydrogenase (GAPDH, AC033), and goat anti-mouse IgG (AS003) were purchased from ABclonal Inc.(Wuhan, China). Taurine bile salt (TCA, T8510) and taurine (T8420) were purchased from Solarbio (Beijing, China). Fibroblast growth factor 15 (FGF15) ELISA Kit was purchased from Uscn Life Science (CEL154Ra, Wuhan, China). 4-kDa fluorescein isothiocyanate (FITC)-dextran was purchased from Sigma (Aldrich, St. Louis, MO, United States). Tachypleus amoebocyte lysate was purchased from Xiamen Bioendo Technology Co., Ltd. (Fujian, China). Carbon tetrachloride, olive oil, and chloral hydrate were purchased from Macklin Biochemical Co., Ltd. (Shanghai, China). All the reagents were of analytical quality.

### Animal study and sample collection

Male Sprague-Dawley (SD) rats at 6 weeks of age and weighing 140-160 g were purchased from the Animal Center of Chongqing Medical University. The rats were housed in standard cages with free access to water and food on a 12:12 h light-dark cycle in a temperature-and humidity-controlled environment. Specific pathogen-free (SPF) rats were randomly allocated to the normal control (NC) and CCl_4_ groups. Liver fibrosis was induced by intraperitoneal administration of CCl_4_ (50% CCl_4_ in olive oil at 1 ml/kg) twice per week for 12 weeks in the CCl_4_ group, while the control group rats received only olive oil (0.5 ml/kg) in the same manner.

**Figure 1:**
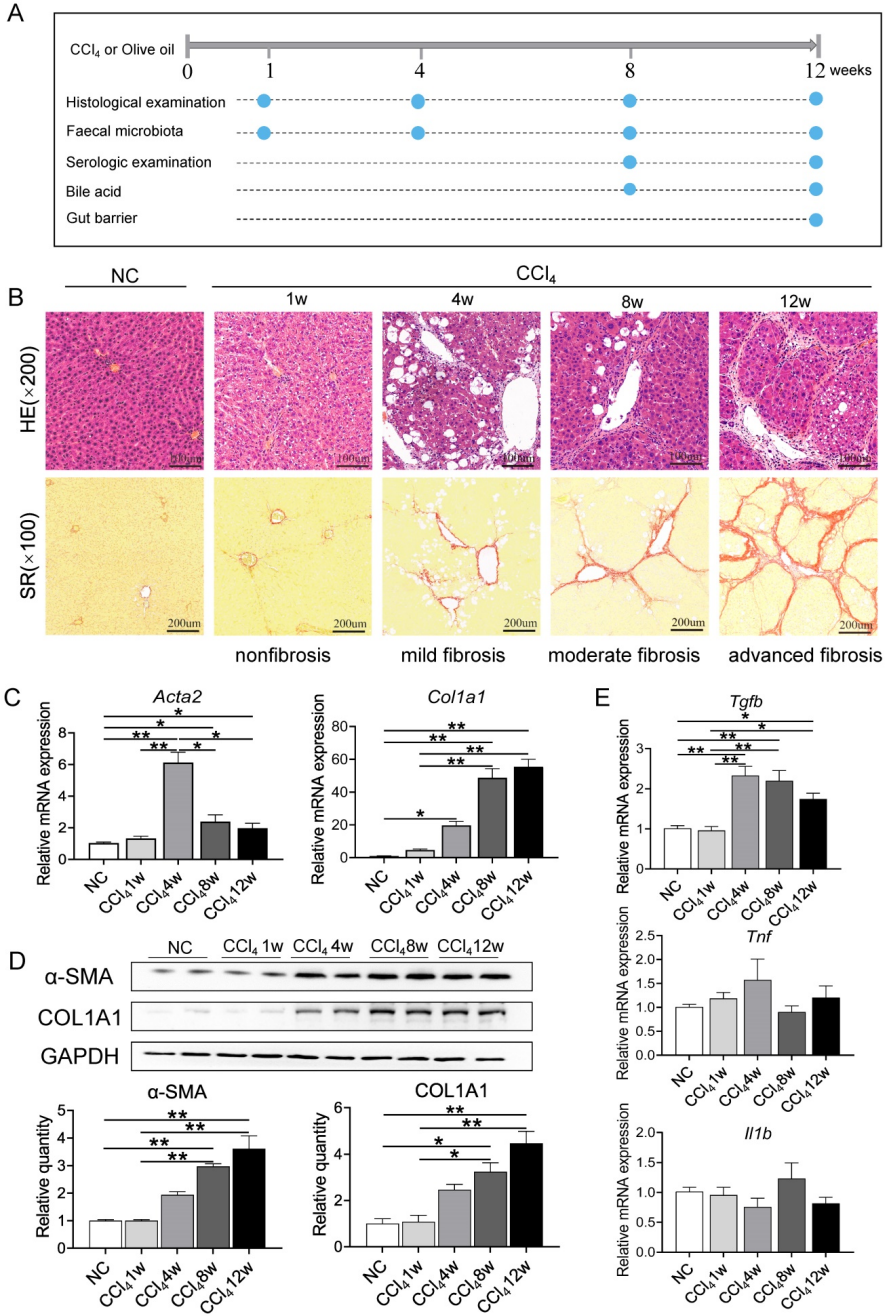
** Liver injury and fibrosis were induced successfully by CCl_4_. (A)** Animal experimental design. **(B)** Representative images of HE staining (original magnification × 200) and SR staining (original magnification × 100) of liver sections from the NC and CCl_4_ groups. **(C-D)** mRNA and protein levels of ACTA2 and COL1A1 in fibrosis progression. **(E)** Gene expression of inflammatory factors in fibrosis progression. Each value is expressed as the mean ± SEM. NC, normal control group; CCl_4_, CCl_4_-treated group; HE, haematoxylin-eosin; SR, Sirius red; Acta2 /α-SMA, actin alpha 2, smooth muscle; *Col1a1*/COL1A1, collagen type I alpha 1 chain; GAPDH, glyceraldehyde-3-phosphate dehydrogenase; *Tgfb*, transforming growth factor beta,* Tnf*, tumor necrosis factor; *Il1b*, interleukin 1 beta. *p<0.05, **p<0.01.

Faecal samples from the same rats were collected at 4 different time points at 1, 4, 8 and 12 weeks. The specimens were gathered at a fixed time point (approximately 9 am) on a clean bench, snap-frozen in liquid nitrogen and then stored at -80 °C until use. Rats were sacrificed by intraperitoneal injection of 10% chloral hydrate (3 ml/kg) mainly at 8 or 12 weeks. The animal experimental design is shown in Figure 1A. All experimental procedures were reviewed and approved by the Chongqing Medical University Ethics Committee.

### Biochemical analysis

Blood was collected by cardiac puncture, and serum was obtained after centrifugation of the blood at 3000 rpm for 10 min at 4 °C. Alanine aminotransferase (ALT), aspartate aminotransferase (AST), albumin (ALB), alkaline phosphatase (ALP), total bile acid (TBa), total bilirubin (TBili), and direct bilirubin (DBili) were detected by a biochemical automatic analyzer at the Clinical Laboratory of the Second Affiliated Hospital of Chongqing Medical University.

### Histopathology

Paraformaldehyde-fixed and paraffin-embedded liver and ileum samples were sectioned and stained with haematoxylin-eosin (HE) to observe lesions, and collagen deposition was detected by staining with Sirius red (SR). The morphological changes were examined by a digital slide scanner and slide viewer software (Pannoramic DESK and Case Viewer 2.4, 3D Histech, Hungary). Liver fibrosis was categorized into 4 stages according to the Ishak score 14: nonfibrosis (Ishak score 0), mild fibrosis (Ishak score 1-2), moderate fibrosis (Ishak score 3-4), and advanced fibrosis (Ishak score 5-6). The integrity of the ileal epithelium was blindly assessed by two senior pathologists following previously described criteria 15.

### Quantitative PCR and Western blot analysis

Total RNA was extracted using an RNA Pure Total RNA Fast Isolation Kit (BioTeke, China) following the manufacturer's instructions. For each sample, 1 μg of total RNA was reverse transcribed using a PrimeScript RT Reagent Kit with gDNA Eraser (TAKARA, Japan) to produce cDNA. Gene expression was measured by qPCR with a CFX Connect™ Real-Time PCR System (Bio-Rad, USA) using the forward and reverse primers shown in Table S1. Data were analyzed using the 2^-ΔΔCt^ method, with GAPDH as the reference marker.

Total protein was extracted from pieces of liver tissues using a whole cell lysis assay (KeyGen Biotech, Jiangsu, China). Protein concentration was determined by the BCA Protein Assay Kit (KeyGen Biotech, Jiangsu, China). Quantified proteins were separated on SDS-PAGE and transferred to PVDF membranes (Millipore Corporation, USA). After blocking by 5% milk, membranes were incubated with primary antibodies against α-SMA (dilution 1:5000), COL1A1 (dilution 1:3000), CYP7A1 (dilution 1:1000), CYP8B1 (dilution 1:1000), FXR (dilution 1:1000), and SHP (dilution 1:1000) at 4 °C overnight. Anti-GAPDH (dilution 1:5000) was probed as an internal control. Then, membranes were washed with TBST and incubated with the secondary antibodies for 2 h at room temperature. Protein bands were visualized by using ECL (Advansta, USA). Levels of target protein band densities were analyzed with a ChemicDoc™ MP Imaging System (Bio-Rad).

### Intestinal microbiota analysis

The intestinal microbiota in 84 stool samples from the 4 time points for the two groups was sequenced at Novogene Co., Ltd. (Beijing, China). DNA was extracted by the cetyltrimethylammonium bromide (CTAB) method, and 16S ribosomal RNA genes were amplified using primers (341F 5'-CCTAYGGGRBGCASCAG-3' and 806R 5'-GGACTACNNGGGTATCTAAT-3') 18 with barcodes. Sequencing libraries were generated using a TruSeq® DNA PCR-Free Sample Preparation Kit (Illumina, USA) following the manufacturer's recommendations. Operational taxonomic units (OTUs) were clustered with a 97% similarity cut-off using Uparse software (Uparse v7.0.1001), and a total of 3588 OTUs, including 2 kingdoms, 20 phyla, 274 genera, and 171 species, were included in the subsequent analysis.

Bacterial richness and diversity within groups were evaluated by α diversity through 4 indexes: Shannon, Simpson, Chao1 and ACE using QIIME (version 1.7.0). Microbial community structure and distribution across groups were evaluated by principal coordinate analysis (PCoA) on weighted UniFrac distance metrics using QIIME software (version 1.9.1), and the difference was calculated by analysis of molecular variance (AMOVA) using mothur (version 1.33.3). Differentially abundant taxa among groups were identified by the linear discriminant analysis (LDA) effect size (LEfSe) method using LEfSe software (version 1.0), and an LDA score > 3 was set as the discrimination threshold.

### Bile acid analysis

Faeces and serum from representative normal rats and CCl_4_-treated rats at the moderate and advanced fibrosis stages were selected for BA determination at Novogene Co., Ltd. (Beijing, China). One hundred milligrams of faeces was resuspended in liquid nitrogen and then added to 0.9 ml of water to prepare a 10-times diluted sample followed by a gradient of 20-times and 200-times dilution. One hundred microliters of serum or pre-diluted faeces suspension was homogenized with 300 or 500 μL of acetonitrile/methanol (8:2) containing mixed internal standards. After centrifugation, protein precipitation, the supernatant was collected and further analyzed for BA concentration using a UPLC-MS/MS system (ExionLC™ AD UHPLC-QTRAP 6500+, AB SCIEX Corp, Boston, MA, USA). Separation was performed on an Agela Venusil MP C18 column (2.1×100 mm, 2.5 μm) at 50 °C. The mobile phase, consisting of 0.1% formic acid in water and acetonitrile, was delivered at a flow rate of 0.50 ml/min. The results are expressed as nanograms of BA per gram of stool and nanograms of BA per milliliter of serum.

### Faecal BSH activity assay

Bile salt hydrolase (BSH) activity was measured by determining the amount of amino acids liberated from conjugated bile salts as previously described with several modifications 16. Briefly, total protein extract was prepared from 50 mg of faecal samples in 250 ul of RIPA by sonication. Protein concentrations were determined with a BCA protein assay kit (keyGEN, Jiangsu, China). Incubation was carried out by adding 10 ul of faecal protein supernatant, 10 ul of TCA (10 mM), and 10ul of paraffin oil to 180 ul of PBS. After a 30 min incubation at 37 °C, reactions were stopped by adding 200ul trichloroacetic acid. Then the mixture was centrifuged and 50 ul of supernatant was added to 950 ul ninhydrin reagent (250 ul of 15% ninhydrin, 600 ul of glycerol, 100 ul of 0.5 M citrate buffer). The preparation was vortexed and boiled for 15 min. After subsequent cooling, the absorbance at 570 nm was determined using taurine as standard.

### FGF15 ELISA

Serum FGF15 was quantified by an ELISA kit following the manufacturer's protocol.

### Intestinal permeability measurement

#### FITC-dextran assay

FITC-dextran levels were measured according to a previously described study, with slight modification 17. Briefly, after rats under anesthesia (10% chloral hydrate 3 ml/kg, intraperitoneally), a midline laparotomy incision was performed, and a 10-cm segment of the distal ileum was isolated between silk ties. A solution of 1 ml of PBS containing 20 mg of 4-kDa FITC-dextran was injected into the lumen of the isolated intestinal segment. The bowel was returned to the abdominal cavity, and the abdomen was covered by sterile gauze, which was wetted with a saline solution at 37 °C. After 30 min, blood was collected by cardiac puncture, and plasma was obtained by centrifugation. Plasma fluorescence was measured at an excitation/emission wavelength of 485 nm/535 nm in a multimode microplate reader to calculate the concentration of FITC-dextran in the blood.

#### Plasma lipopolysaccharide (LPS) assay

Plasma LPS quantification was performed using tachypleus amoebocyte lysate according to the manufacturer's protocol. Briefly, blood was placed in endotoxin removal heparinized Eppendorf tubes and centrifuged at 3000 rpm for 2 min at 4 °C. Next, 0.1 ml of plasma supernatant was added to 0.9 ml of blood sample treatment fluid. Then, a standard solution was prepared and diluted to 0.1, 0.05, 0.025 and 0.01 EU/mL. Next, tachypleus amoebocyte lysate was added, and the sample was vortexed and then incubated at 37 °C for 35 min. Subsequently, the chromogenic substrate was added, and the sample was vortexed and incubated at 37 °C for 6 min. Finally, azo reagent was added, the sample was vortexed and allowed to stand for 5 min, and the absorbance was read at 545 nm.

### Statistical analysis

Data are expressed as the mean ± SEM unless otherwise stated. Statistical analyses were performed by SPSS (IBM SPSS 26.0) between groups and displayed by GraphPad Prism (version 8.0.2.). The dynamic alteration of the gut microbiota among the 4 fibrosis stages was analyzed by the Friedman test. The correlations between the relative abundance of the microbiome constituents and ALT, AST and ALP levels were evaluated by Spearman's test. Comparisons of 16S rDNA sequencing and BA analysis not mentioned above were conducted using nonparametric statistics. Other parameters, such as body weight, biological indicators, and mRNA expression with a normal distribution and homogeneity of variance, were evaluated by Student's t test; otherwise, they were evaluated by the Mann-Whitney U test. P values were corrected by the Benjamini-Hochberg method for multiple comparisons, and P<0.05 was considered statistically significant.

## Results

### Characteristics of CCl_4_-induced liver fibrosis

With respect to the characterization of biochemical and histological changes associated with injury, inflammation, and fibrosis, CCl_4_-induced liver fibrosis and cirrhosis models mirror the pattern of human fibrosis and cirrhosis caused by toxic damage 12, 13. As shown in Figure 1A, rats either received twice-weekly intraperitoneal injection of CCl_4_ or olive oil alone as a control for 12 weeks. Intoxication with CCl_4_ resulted in slower weight gain of rats in the model group at each sampling point (Figure S1A). The ratio of liver mass to body weight was significantly higher in CCl_4_-administered rats than in control rats at week 8 and week 12 (Figure S1B). In addition, ALT, AST, ALP activity and TBa, DBili, and TBili levels in the CCl_4_ groups were significantly increased compared to control groups, and CCl_4_-treated rats had a lower serum ALB level than the control rats at the two sampling points (Figure S1C).

Liver samples were stained with HE and SR to evaluate histopathologic changes and tissue fibrosis. Figure 1B shows that liver tissue from control rat exhibited normal cellular architecture and normal collagen distribution. Liver specimens from rats injected with CCl_4_ for 1, 4, 8 and 12 weeks exhibited different pathological changes and fibrosis degrees. Liver tissue from the CCl_4_ group at week 1 (nonfibrosis, Ishak score 0) demonstrated some cellular damage and centrilobular congestion with no infiltration of inflammatory cells. Repetitive dosing of CCl_4_ resulted in hepatocytic fatty degeneration and extension of fibres in the central vein and portal area at week 4 (mild fibrosis, Ishak score 1-2), bridging hepatic fibrosis at week 8 (moderate fibrosis, Ishak score 3-4) and extensive collagen deposition and pseudolobular formation at week 12 (advanced fibrosis/cirrhosis, Ishak score 5-6). The severity of fibrosis was positively associated with further CCl_4_ injections, progressing from no obvious fibre deposition at week 1 to nodule formation or cirrhosis at week 12.

To further confirm the degree of fibrosis, expression levels of genes related to hepatic stellate cells (HSCs) activation (actin alpha 2, smooth muscle,* Acta2*; transforming growth factor beta cytokines, *Tgfb*; tumor necrosis factor, *Tnf*; interleukin 1 beta,* Il1b*) and ECM production (collagen type I alpha 1 chain, *Col1a1*) and protein levels of α-SMA and COL1A1 were detected during liver fibrosis. Compared to normal control and nonfibrosis condition, *Acta2* was significantly upregulated since week 4 (mild fibrosis stage), with a consistent trend of change with *Tgfb* (Figures 1C, 1E). Repeated administration of CCl_4_ caused a gradual elevation of *Col1a1* mRNA expression and protein levels of COL1A1 and α-SMA, confirming the progression of liver fibrosis (Figure 1D). However, there was no significant difference in the gene expression of inflammatory factors *Tnf* and *Il1b* between the normal control group and CCl_4_-treated groups (Figure 1E).

### Gut microbiota profile at different fibrosis stages

**Figure 2:**
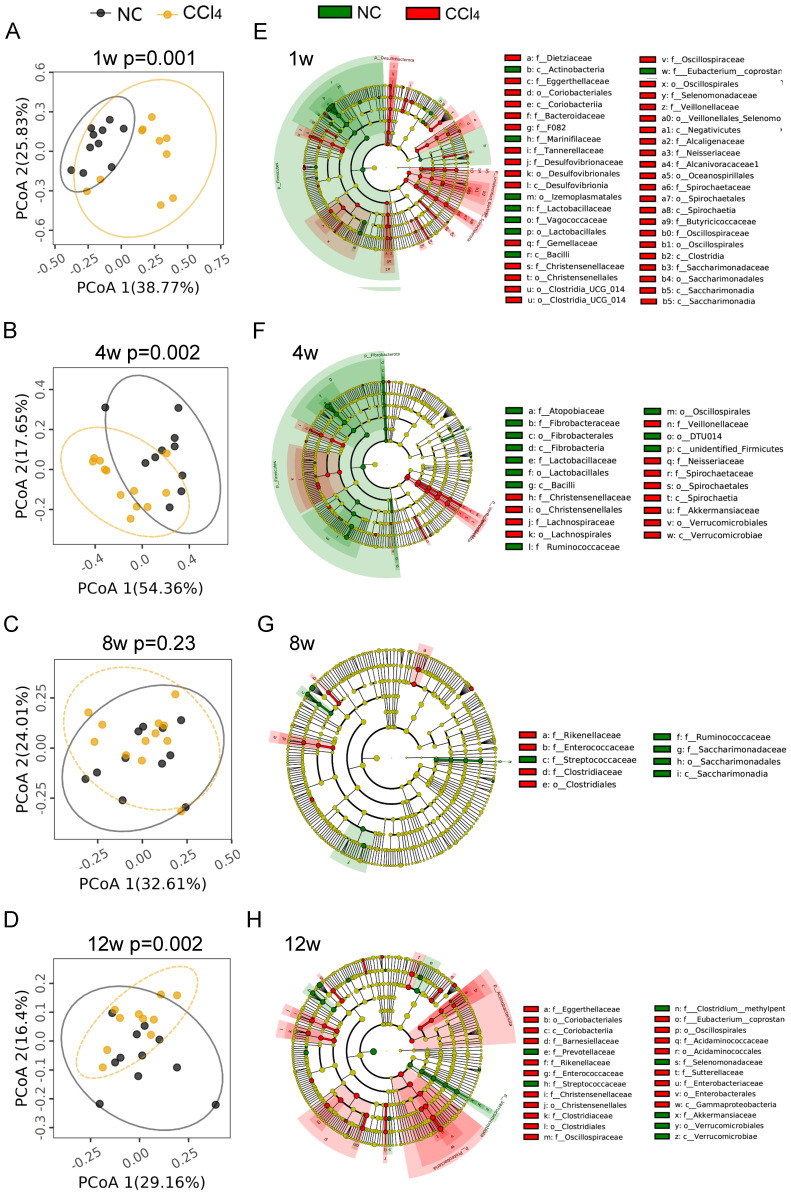
** Comparison of gut microbiota compositions between the normal control groups and fibrosis groups. (A-D)** PCoA plots of microbial communities at weeks 1, 4, 8 and 12. PCoA based on weighted_UniFrac distance was tested with AMOVA, and the samples at week 8 were mixed together, while obvious differences were observed at weeks 1, 4 and 12 between the two groups (n=10-12). **(E-H)** Cladogram exhibiting distinctive gut microbiome profiles from the phylum to the species level from inside to outside. Yellow circles represent taxa that did not differ between the two groups. Green and red circles represent distinctive microbiota constituents in the normal control and CCl_4_ groups, respectively. The size of the circle depicts the relative abundance. p_, phylum; c_, class; o_, order; f_, family.

To explore the alteration of the gut microbiota at different fibrosis stages, we compared the gut microbiota composition between the two groups at the same time points. PCoA plots showed that samples with nonfibrosis, mild fibrosis and advanced fibrosis were separated from their corresponding normal controls (Figures 2A-2B, 2D, Table S2), while samples with moderate fibrosis mixed together with normal control samples (Figure 2C, Table S2, AMOVA p=0.23).

Although there was no difference in gut microbial diversity or richness between the control and the liver injury groups at the four different fibrosis stages (Figures S2A-B), the abundance of gut microbiota at different phylogenetic levels remained significantly different between the two groups. As shown in Figures 2E-2H, microbial cladograms exhibited distinctive gut microbiome profiles from the phylum to the species levels at the 4 fibrosis stages. Before the formation of liver fibrosis (nonfibrosis), the most significant changes in gut microbiota were in *Desulfovibrio* (Desulfobacterota), *Treponema* (Spirochaetota) and *Lactobacillus* (Firmicutes), with changes from the genus level to the phylum level. Among them, the relative abundance of genera *Desulfobacterota* and *Treponema* increased in the liver injury group, and the relative abundance of *Lactobacillus* decreased (Figure 2E, Table S3). At the mild fibrosis stage, the relative abundance of *Treponema* and *Akkermansia* (Verrucomicrobiota) significantly increased, while the relative abundance of *Lactobacillus* and *Fibrobacter* (Fibrobacterota) decreased (Figure 2F, Table S3). For the moderate fibrosis stage, genera *Rikenellaceae_RC9_gut_group*, *Clostridium_sensu_stricto_1*, *Enterococcus* and *Lachnospiraceae_UCG_010* were increased, while *Streptococcus*, *Ruminococcus* and *Candidatus_Saccharimonas* were decreased (Figure 2G, Table S3). At this stage, gut microbiota differences were mainly noted at low phylogenetic levels like genus and species, indicating that gut dysbiosis was alleviated. At the advanced fibrosis stage, Actinobacteriota, Proteobacteria and Verrucomicrobiota were the most changed taxa (Figure 2H, Table S3). The increase in the abundance of potentially pathogenic *Enterobacteriaceae* (Proteobacteria) and *Eggerthellaceae* (Actinobacteriota) and the decrease in the abundance of beneficial *Akkermansiaceae* (Verrucomicrobiota) implicated that dysbiosis deteriorated again. As liver fibrosis progressed, the profile of the dysregulated microbiota varied generally, but Lactobacillus at different phylogenetic levels were consistently underrepresented in fibrosis groups (Figures 2E-2H, Table S3). The relative abundance of *Lactobacillus_murinus* was reduced over the four fibrosis stages, other Lactobacillus species like *Lactobacillus_intestinalis*, *Lactobacillus_johnsonii*, *Lactobacillus_reuteri*, *Lactobacillus_sp_KC38* and *Lactobacillus_faecis* was significantly reduced at the nonfibrosis and/or mild fibrosis stages (Table S3).

### Dynamics of the characteristic gut microbiome over the evolution of liver fibrosis

To investigate the temporal development of the gut microbiota in the process of liver fibrosis, faecal samples from 4 different time points (weeks 1, 4, 8 and 12; equivalent to 7, 10, 14, and 18 weeks of age in rats) were analyzed. PCoA plots showed that samples were basically clustered together at different time points, but differences still existed between any two time points (Figure S2C, Table S2), suggesting that gut microbiome community in the same environment changes slightly during development. The gut microbiota at week 1 in the CCl_4_ group showed high variability, reflecting gut microbiota susceptibility to short-term environment exposure (Figure S2C). In normal rats, gut microbiota diversity and richness continued to increase with age, peaking at approximately 14 weeks of age (at week 8) and then remaining stable (Figure S2D). However, in the presence of liver fibrosis, gut microbiota diversity increased with age, and the diversity at 18 weeks (week 12) was significantly higher than the previous three time points, indicating a delay in community stability (Figure S2D). Gut microbiota richness remained relatively stable in CCl_4_-treated rats compared to that in normal rats (Figure S2E).

**Figure 3:**
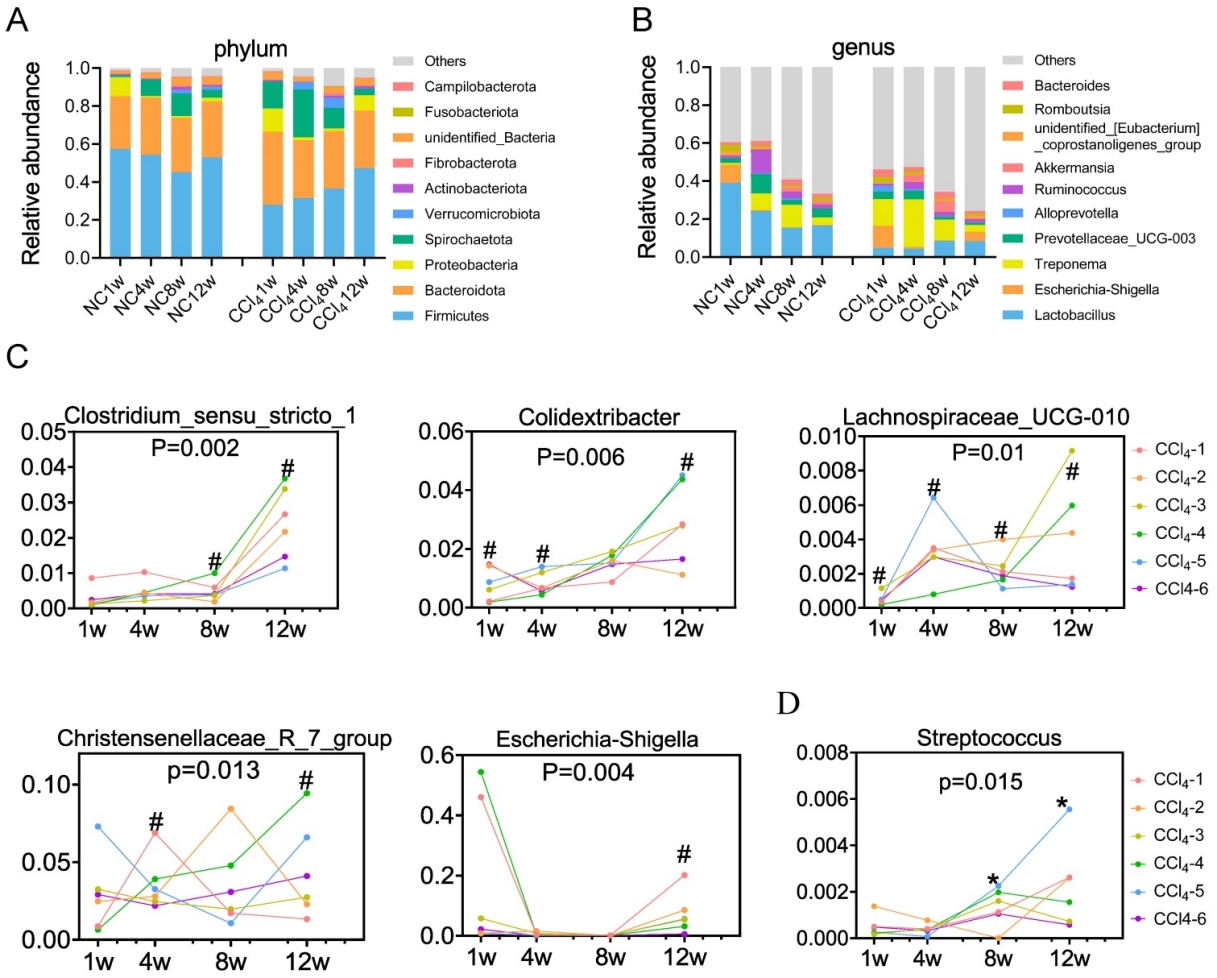
** Temporal development of the gut microbiota in the normal control and the fibrosis groups. (A)** Temporal development of the major phyla (n=10-12). **(B)** Temporal development of the major genera (n=10-12). **(C)** Temporal development of distinctive gut microbiota (overrepresented in liver injury groups) over liver fibrosis (n=6). **(D)** Temporal development of distinctive gut microbiota (underrepresented in liver injury groups) over liver fibrosis (n=6). The same color represents the same numbered rat # the genus was increased in the fibrosis group, * the genus was decreased in the fibrosis group compared to that in the normal control group. The alteration of the gut microbiota among the four fibrosis stages was analyzed by the Friedman test.

At the phylum level, both the control group and CCl_4_ group were dominated by Firmicutes (means of 52% and 36%, respectively), Bacteroidota (29% and 32%, respectively), Spirochaetota (7% and 14%, respectively) and Proteobacteria (3% and 5%, respectively) (Figure 3A, Table S4). Firmicutes and Proteobacteria exhibited distinctive temporal changes in the relative abundance between the two groups. The relative abundance of Firmicutes remained relatively stable in control rats over time, whereas it increased in CCl_4_-treated rats throughout the progression of liver fibrosis. The relative abundance of Proteobacteria was higher at week 1 in normal rats but higher at nonfibrosis and advanced fibrosis stages in CCl_4_-treated rats (Figure 3A, Table S4). At the genus level, the relative abundance of *Lactobacillus* showed a decreasing trend in the normal control groups, while that showed an increasing trend in the CCl_4_ treatment groups. Although the relative abundance of *Lactobacillus* attempted to increase under fibrosis conditions, it was consistently lower than that under normal conditions (Figure 3B, Table S5). Changes in the relative abundance of other phyla and genera were shown in Table S4 and Table S5.

Compared to those in the control group, genera *Clostridium_sensu_stricto_1, Colidextribacter*, *Lachnospiraceae_UCG_010, Christensenellaceae_R_7_group* and *Escherichia_Shigella* were generally overrepresented in the liver injury group, further enriched in the advanced fibrosis stage (Figure 3C). *Streptococcus* was underrepresented in the moderate and advanced fibrosis stages, despite an increasing trend from the mild fibrosis to the advanced fibrosis stage (Figure 3D). Spearman correlation tests demonstrated that relative abundances of *Clostridium_sensu_stricto_1*, *Escherichia_Shigella* and *Lachnospiraceae_UCG_010* were positively associated with serum ALT, AST and ALP levels (Figure S3A). Relative abundances of *Christensenellaceae_R_7_group* and *Colidextribacter* were positively associated with the ALT level (Figure S3A). Relative abundance of *Streptococcus* was found to be negatively associated with serum ALT, AST and ALP levels (Figure S3B).

### Faecal unconjugated BAs decreased and serum conjugated BAs increased in liver fibrosis progression

**Figure 4:**
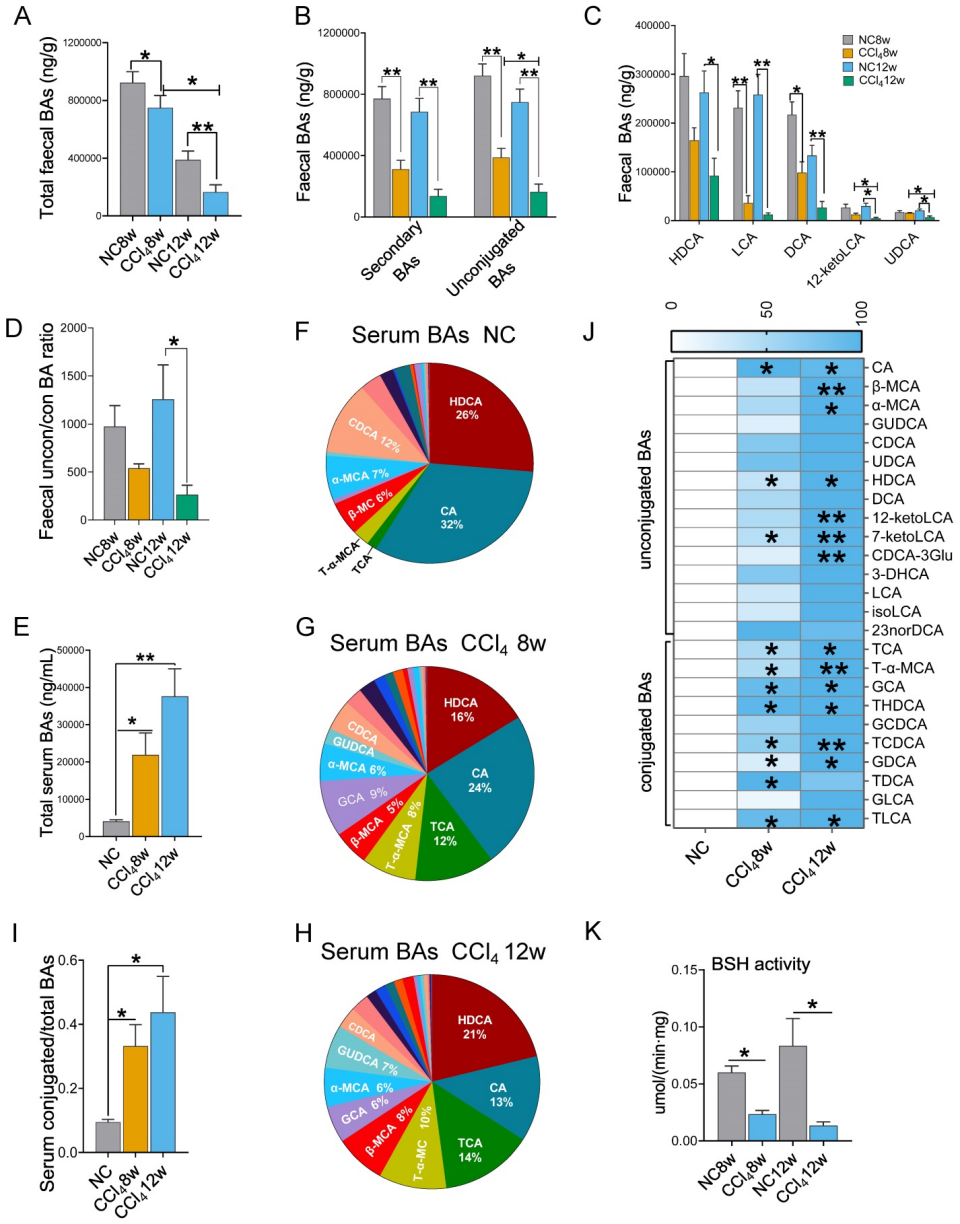
** Faecal and serum BA profiles (faecal n=6 per group, serum n=4 per group). (A)** Faecal total BA levels. **(B)** Faecal secondary BA and unconjugated BA levels. **(C)** HDCA, LCA, DCA, 12-ketoLCA, and UDCA decreased significantly in the liver fibrosis groups. **(D)** The ratio of unconjugated BAs to conjugated BAs in faeces. **(E)** Serum total BA levels. **(F-H)** Composition of serum BAs in the normal control, the moderate fibrosis stage, and the advanced fibrosis stage. **(I)** The ratio of conjugated BAs to total BAs in serum. **(J)** Comparison of individual BA between the normal control group and the fibrosis groups, * representing difference compared to the normal control group. **(K)** Faecal BSH activity. Data are expressed as the mean ± SEM. BA(s), bile acid(s); uncon/con BAs, unconjugated BAs/conjugated BAs; CA, cholic acid; β-MCA, beta-muricholic acid; α-MCA, alpha-muricholic acid; GUDCA, glycoursodeoxycholic acid; CDCA, chenodeoxycholic acid; UDCA, ursodeoxycholic acid; HDCA, hyodeoxycholic acid; DCA, deoxycholic acid; 12-ketoLCA, 12-ketolithocholic acid; 7-ketoLCA, 7-ketolithocholic acid; CDCA-3Glu, chenodeoxycholic acid-3-β-D-glucuronide; 3-DHCA, 3-dehydrocholic acid; LCA, lithocholic acid; isoLCA, isolithocholic acid; TCA, taurocholic acid; T-α-MCA, tauro-alpha-muricholic acid; GCA, glycocholic acid hydrate; THDCA, taurohyodeoxycholic acid; GCDCA, glycochenodeoxycholic acid; TCDCA, taurochenodeoxycholic acid; GDCA, glycodeoxycholic acid; TDCA, taurodeoxycholic acid; GLCA, glycolithocholic acid; TLCA, taurolithocholic acid; BSH, bile salt hydrolase. *p<0.05, **p<0.01.

BAs are the link between the gut and the liver and play important roles in bidirectional communication. We detected BAs in faeces and serum at the moderate and advanced hepatic fibrosis stages. With the progression of liver fibrosis, the total BA level in faeces decreased, while that in serum increased (Figures 4A, 4E). Advanced fibrosis resulted in the lowest faecal secondary BA levels, unconjugated BA levels and unconjugated/conjugated BA ratio compared to those in the moderate fibrosis and the control condition (Figures 4B, 4D). Among individual BAs, lithocholic acid (LCA), deoxycholic acid (DCA), hyodeoxycholic acid (HDCA), 12-ketolithocholic acid (12-ketolLCA) and ursodeoxycholic acid (UDCA) were significantly reduced in faeces with the progression of liver fibrosis (Figure 4C).

Serum BAs were dominated by unconjugated BAs (Figures 4F-4H). However, in the presence of liver fibrosis, the ratio of conjugated BAs increased (Figure 4I), among which tauro-alpha-muricholic acid (T-α-MCA) and taurocholic acid (TCA) revealed the most significant elevations (Figures 4F-4H)). As fibrosis aggravated, all serum levels of individual BAs tended to elevate, with a particularly significant elevation in conjugated BAs (Figure 4J).

BSH is responsible for converting conjugated BAs to unconjugated BAs in the intestine 18. We found that BSH activity was significantly decreased in fibrosis groups (Figure 4K). This result was consistent with the changes in BA profile: a decrease in unconjugated BAs in faeces and an increase in conjugated BAs in serum.

### Regulation of BAs by enterohepatic circulation during fibrosis

**Figure 5:**
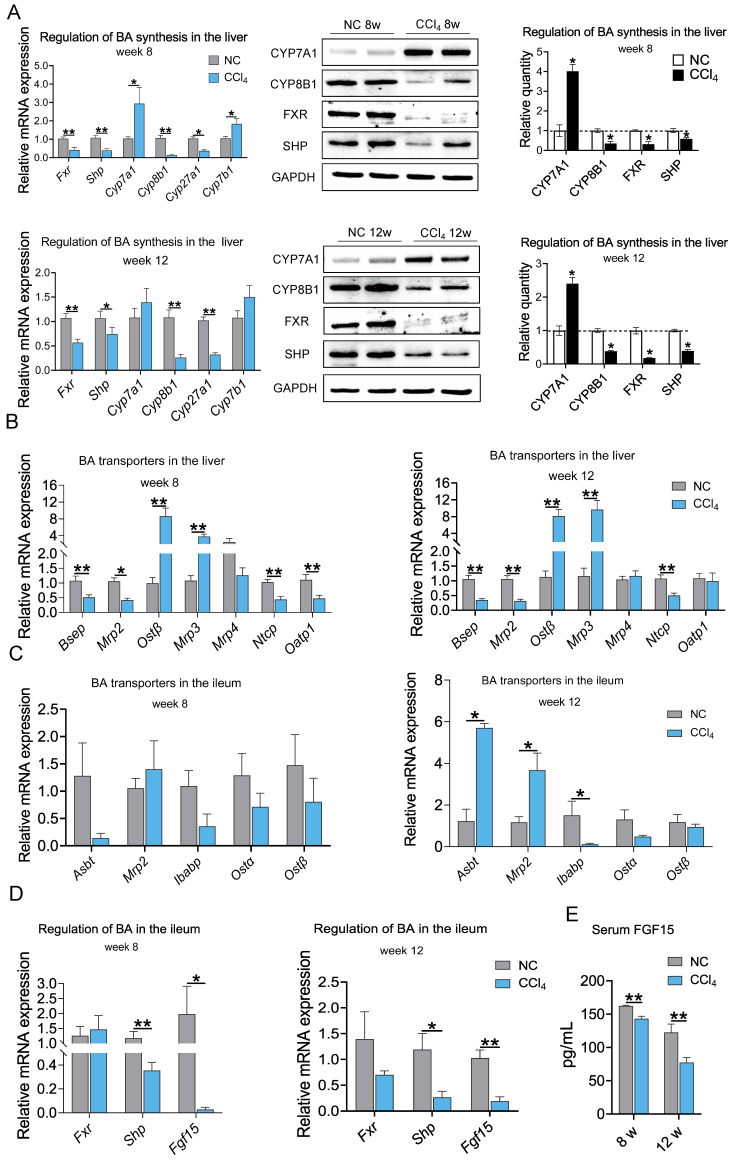
** Protein and mRNA expression associated with BA synthesis and BA transporters at weeks 8 and 12 in the liver and ileum** (protein n=4, mRNA n=6-8). **(A)** Regulation of BA synthesis in the liver. **(B-C)** mRNA expression of BA transporters in the liver and ileum. **(D)** mRNA expression of *Fxr*, *Shp* and *Fgf15* in the ileum. **(E)** Serum FGF15 protein level. Data are expressed as the mean ± SEM. *Fxr* /FXR, farnesoid X receptor; *Shp*/SHP, small heterodimer partner; *Cyp7a1*/CYP7A1, cytochrome P450 family 7 subfamily A member 1; *Cyp8b1*/CYP8B1, cytochrome P450 family 8 subfamily B member 1;* Cyp27a1*, cytochrome P450, family 27, subfamily a, polypeptide 1;* Cyp7b1*, cytochrome P450 family 7 subfamily B member 1; *Bsep*, bile-salt export pump; *Mrp2*, multidrug-resistance-associated protein 2; *Ibabp*, ileal BA binding protein; *Ostα/β*, Organic solute transporter subunit α/β; *Fgf15*/FGF15, fibroblast growth factor 15. *p<0.05, **p<0.01.

To further explore the mechanism of the changes in BA levels across fibrosis severities, we evaluated molecular changes in BA synthesis and regulation at the gene and protein levels. The mRNA expression levels of key enzymes in classical or alternative BA synthesis pathways,* Cyp7a1* and cytochrome P450 family 7 subfamily B member 1 (*Cyp7b1*), were significantly increased in the moderate liver fibrosis stage and tended to increase in the advanced fibrosis stage. The mRNA levels of *Cyp8b1* and cytochrome P450, family 27, subfamily a, polypeptide 1 (*Cyp27a1*) were significantly decreased in the fibrosis groups. Hepatic mRNA expression of *Fxr* and *Shp* was significantly downregulated in rats with moderate and advanced fibrosis. Consistent with the gene expression trend, higher CYP7A1 and lower CYP8B1, FXR, and SHP protein levels were observed in the liver fibrosis groups (Figure 5A). These results indicate that BA synthesis was increased in liver fibrosis.

In addition to BA biosynthesis, a variety of hepatic and intestinal membrane transporters play critical roles in maintaining BA homeostasis. Hepatic mRNA expression levels of bile-salt export pump (*Bsep*), multidrug-resistance-associated protein 2 (*Mrp2*), and Na^+^-taurocholate cotransporting protein (*Ntcp*) were significantly decreased in both the moderate and advanced fibrosis groups. mRNA expression of organic anion-transporting polypeptide 1 (*Oatp1*) was decreased in moderate fibrosis but showed no difference in advanced fibrosis. Organic solute transporter subunit β (*Ostβ*) and *Mrp3* mRNA levels were increased, while the mRNA level of *Mrp4* was not altered in liver fibrosis conditions (Figure 5B). Down-regulation of genes responsible for the excretion of BAs into the bile canaliculi (*Bsep* and* Mrp2*) and the recycling of BAs to the liver (*Ntcp* and *Oatp1*) and up-regulation of genes responsible for the excretion of BAs into the circulation (*Ostβ* and* Mrp3*) resulted in a decrease in BAs excretion into the intestinal and an increase in BAs entering the circulation. At the moderate fibrosis stage, the mRNA levels of ileal BA transporters, including sodium-dependent BA transporter (*Asbt*), *Mrp2*, ileal BA binding protein (*Ibabp*), *Ostα* and *Ostβ*, were not significantly changed. *Asbt* and *Mrp2* expression was increased, and *Ibabp* expression was decreased significantly in advanced fibrosis (Figure 5C). The ileal FXR-FGF15 pathway was suppressed with downregulation of *Shp* and *Fgf15* expression (Figure 5D), which resulted in a significant reduction in circulating FGF15 protein levels in the fibrosis groups (Figure 5E). These results suggest that BA synthesis increased while BA excretion into the intestine decreased during hepatic fibrosis.

### The gut barrier remained intact in advanced hepatic fibrosis

**Figure 6:**
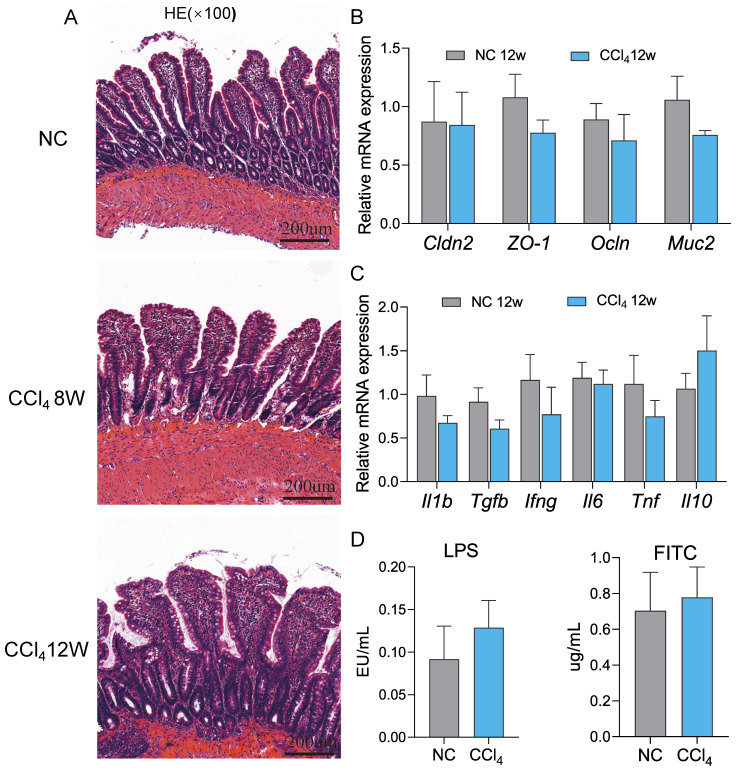
** Assessment of ileal permeability at the advanced fibrosis stage.** (A) Representative images of HE staining (original magnification × 100) of the ileum in the normal control, moderate and advanced fibrosis groups. (B-C) Gene expression of barrier proteins and inflammatory factors in the ileum (n=4-6). (D) Plasma FITC and LPS levels (n=6). Each value is expressed as the mean ± SEM. *Cldn2*, claudin-2; *ZO-1*, zonula occludens1; *Ocln*, occludin; *Muc2*, mucin 2; LPS, lipopolysaccharides; FITC, fluorescein isothiocyanate-dextra.

Dysfunction of the physical barrier contributes to the development of diseases in the liver 19, and we evaluated the integrity of the ileal epithelium in the advanced fibrosis group. No significant degeneration of epithelial cells, villus blunting or submucosal inflammation in terminal ileum histology were observed in the moderate and advanced fibrosis groups compared to the normal control group (Figure 6A). In accordance with the histology results, the expression of the typical tight junctions of claudin-2 (*Cldn2*), zonula occludens1 (*ZO-1*), and occludin (*Ocln*) and small intestinal mucus protein mucin 2 (*Muc2*) at the mRNA levels remained unchanged in the advanced fibrosis group (Figure 6B). Similarly, the mRNA levels of the inflammatory factors *Il1b*, *Tgfb*, interferon gamma (*Ifng*), interleukin 6 (*Il6*), *Tnf* and interleukin 10 (*Il10*) also did not differ significantly between the control group and advanced fibrosis group (Figure 6C). Although the plasma LPS and FITC levels in the advanced fibrosis group tended to increase, the difference was not statistically significant (Figure 6D). Gene expression of inflammatory factors and barrier proteins in the colon also showed the same trend as that in the ileum (Figure S4). These results implied that the gut barrier remained relatively intact in rats with advanced fibrosis induced by intraperitoneal injection of CCl_4_ in our experiment settings.

## Discussion

This study investigated the dynamic changes in the gut microbiota, BAs and the gut epithelial barrier across liver fibrosis severities in CCl_4_-induced rats. We found that the gut microbiota profile changed as fibrosis progressed but was not necessarily positively correlated with the fibrosis degree. As fibrosis progressed, the level of unconjugated BAs in faeces gradually decreased, while the level of conjugated BAs in serum gradually increased. In our experiment settings, we found that the intestinal barrier remained relatively structurally and functionally intact even in advanced liver fibrosis.

Longitudinal study of the faecal microbiota demonstrated that gut microbiota dysbiosis was more severe in the prophase of fibrosis and advanced fibrosis stage than in the mild and moderate fibrosis stages in the CCl_4_-induced rat model. Opportunistic pathogens such as the taxa *Desulfovibrio*, *Treponema* and* Eggerthellaceae* were significantly overrepresented, while beneficial commensals such as the taxa *Lactobacillus* and *Akkermansia* were obviously underrepresented at the early and advanced stages of fibrosis. *Desulfovibrio* and *Eggerthellaceae* are thought to promote intestinal inflammation and are widely involved in the aetiology of gastrointestinal diseases 20, 21. The taxon *Enterobacteriaceae* has long been considered as a potential diagnostic signature of dysbiosis 22 and is markedly increased in cirrhosis 23. Healthy promotion of autochthonous *Lactobacillus* and *Akkermansia* proliferation plays essential roles in protecting against pathogens and maintaining gut barrier integrity 24-26. These unfavourable shifts in the gut microbiota may contribute to the initiation and progression of liver fibrosis. In the mild fibrosis stage, there was a compensatory increase in the abundances of beneficial taxa, such as Akkermansia, despite the continuous increase in the abundance of pathogenic bacteria. Gut dysbiosis was alleviated visibly in the moderate fibrosis stage, when the differences in the microbiota between the control group and moderate fibrosis group mainly occurred at the species and genus levels. These results suggest that the disturbed microbiota is capable of reattaining homeostasis to some extent before advanced fibrosis; thus, gut dysbiosis may not parallel the degree of liver fibrosis.

Lactobacillus at different phylogenetic levels was reduced in the fibrosis groups compared to that in their corresponding normal control groups, among which the species *Lactobacillus_murinus* was the most significantly affected species, showing a declining trend in all the 4 fibrosis stages. Indeed, *Lactobacillus* has been widely used as probiotics with beneficial effects on liver injury and ameliorating liver fibrosis 27, 28. Liu Y *et al.* has further demonstrated that Lactobacillus supplementation prevents liver fibrosis by inhibiting liver BA synthesis through intestinal FXR-FGF15 signalling. Meanwhile, Lactobacillus treatment increases faecal BA excretion through increasing BSH-containing gut bacteria and promotes urine BA excretion through an FXR-independent pathway 29. Our results further support the view that *Lactobacillus* may be a distinctive gut biomarker for liver fibrosis and a new target to fight fibrosis.

Most of the differentially abundant gut microbiota constituents remained stable across the development of liver fibrosis; however, the minority of taxa changed in abundance with fibrosis progression. *Streptococcus* abundance showed an increasing trend at the moderate and advanced fibrosis stages, but its abundance was still significantly lower than that in the control group. Relative abundance of *Streptococcus* was negatively associated with serum ALT, AST and ALP levels, indicating that the decrease in *Streptococcus* abundance is an adverse change in liver injury. *Clostridium_sensu_stricto_1*, *Escherichia_Shigella*, *Lachnospiraceae_UCG_010*, *Christensenellaceae_R_7_group*, and *Colidextribacter* were more abundant in the advanced fibrosis stage than in the early and middle stages. *Christensenellaceae_R_7_group* was reported to be beneficial to the intestinal barrier 30, 31, while other gut bacteria have been reported to be involved in intestinal inflammation 32-34 and a variety of liver disorders, including cirrhosis 35, 36. Moreover, we found a positive correlation of the relative abundances of *Clostridium_sensu_stricto_1*, *Escherichia_Shigella*, and *Lachnospiraceae_UCG_010* with serum ALT, AST, and ALP levels and of the relative abundances of *Christensenellaceae_R_7_group* and *Colidextribacter* with ALT levels. The increase in the abundance of these microbiota may directly injure the liver and promote the occurrence of advanced fibrosis and even the subsequent progression of cirrhosis.

Previous studies have demonstrated that reductions in faecal total BA, secondary BA and unconjugated BA levels parallel the severity of liver fibrosis 37, 38. Consistently, the concentrations of faecal total BAs, secondary BAs and unconjugated BAs decreased progressively across the normal control condition, moderate fibrosis and advanced fibrosis in our study. Firstly, the hepatic FXR-SHP and intestinal FXR-FGF15 pathways are the primary regulators of BA biosynthesis in enterohepatic circulation. Reduced unconjugated BAs in faeces of rats with advanced fibrosis are mainly LCA and DCA, which are agonists of intestinal FXR 39. The elevated conjugated BAs in serum were dominated by TCA and T-α-MCA, of which T-α-MCA is an antagonist of FXR 40. Decreases in LCA and DCA and an increase in T-α-MCA resulted in a significant downregulation of serum FGF15, which ultimately resulted in an increase in hepatic BA synthesis. Secondly, the reduced BSH activity led to the decrease in unconjugated BA levels in faeces and the increase conjugated BA levels in serum from another perspective. Because Firmicutes is rich in BSH 41 and genus *Lactobacillus* particularly possesses strong BSH activity 42, the reduction in Firmicutes abundance along with that of its affiliated families *Lactobacillaceae* and *Ruminococcaceae*, and genera *Lactobacillus* and *Ruminococcus* may account for the decrease in BSH activity. Therefore, adverse changes in the gut microbiota altered the BA profiles, which in turn inhibited FXR signalling pathway, ultimately resulting in BA accumulation. An excess of cytotoxic BAs in the liver can lead to liver fibrosis and cirrhosis.

Gut barrier is reported to be disrupted during liver fibrosis 43-46. However, we did not observe significant gut barrier disruption in our study. Du Plessis J *et al.* had reported that the intestinal barrier could remain intact in patients with compensatory cirrhosis until decompensated cirrhosis occurred 47, suggesting that the severe liver fibrosis stage is more likely to be accompanied by intestinal barrier disruption. Although we did not detect significant intestinal barrier disruption from multiple perspectives in our study, we still observed a trend of increased intestinal permeability to LPS and FITC at the advanced liver fibrosis stage. Therefore, our results do not contradict previous studies, and the differences may lie in the degree of fibrosis.

Taken together, the main results of the present work are the characterization of the dynamics of the gut-liver axis in the progression of liver fibrosis. To the best of our knowledge, this study is the first attempt to elucidate temporal variation in the gut microbiota constituents and BA levels in the CCl_4_-induced fibrosis model, which can provide the basis for further mechanistic and interventional studies to prevent or reverse the liver fibrosis phenotype. However, the causal relationship of the gut microbiota, BAs and liver fibrosis is still obscure, and further investigation is needed to clarify the mechanisms involved.

## Supplementary Material

Supplementary figures and tables.
