# Supplementary material for: Dynamics of the gut-liver axis in rats with varying fibrosis severity
Source: Int J Biol Sci. 2022 May 9;18(8):3390–404. doi: 10.7150/ijbs.69833 (PMC9134918; doi:10.7150/ijbs.69833)
Supplement: Supplementary file 1 — Supplementary figures and tables. [file ijbsv18p3390s1.pdf]

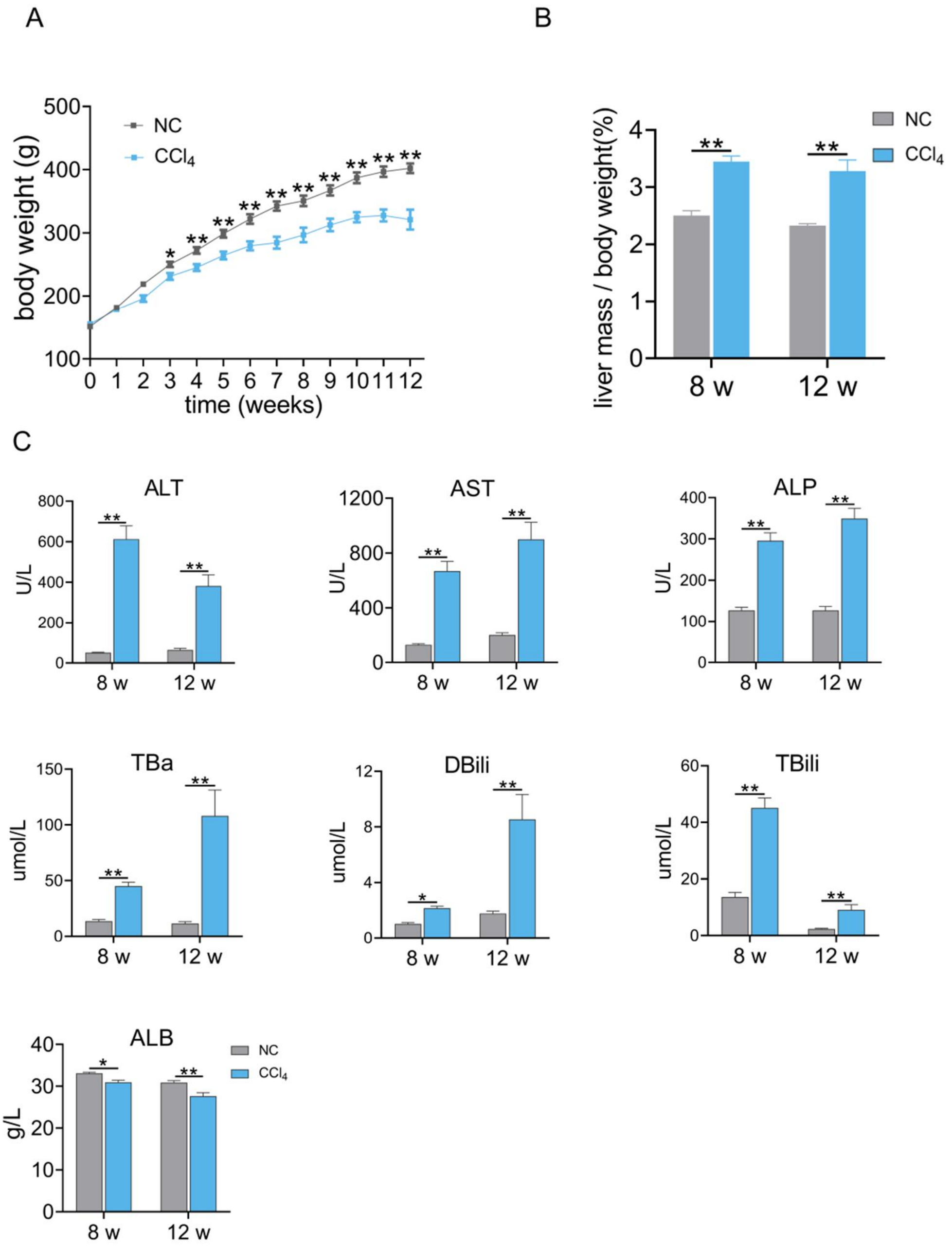

Figure S1. Liver injury and fibrosis were induced successfully by CCl_4. (A) Body weight of rats over the whole experimental period (n=12 per group). (B) Liver mass/ body weight (n=12 per group). (C) Serum ALT, AST, ALP, TBa , DBili, TBili, and ALB levels (8w n=10,12w n=12). Each value is expressed as the mean ± SEM. NC, normal control group; CCl_4, CCl_4-treated group; ALT, alanine transaminase; AST, aspartate aminotransferase; ALP, alkaline phosphatase; TBa, total bile acid; DBili, direct bilirubin; TBili, total bilirubin; ALB, albumin. *$p<0.05$, **$p<0.01$.

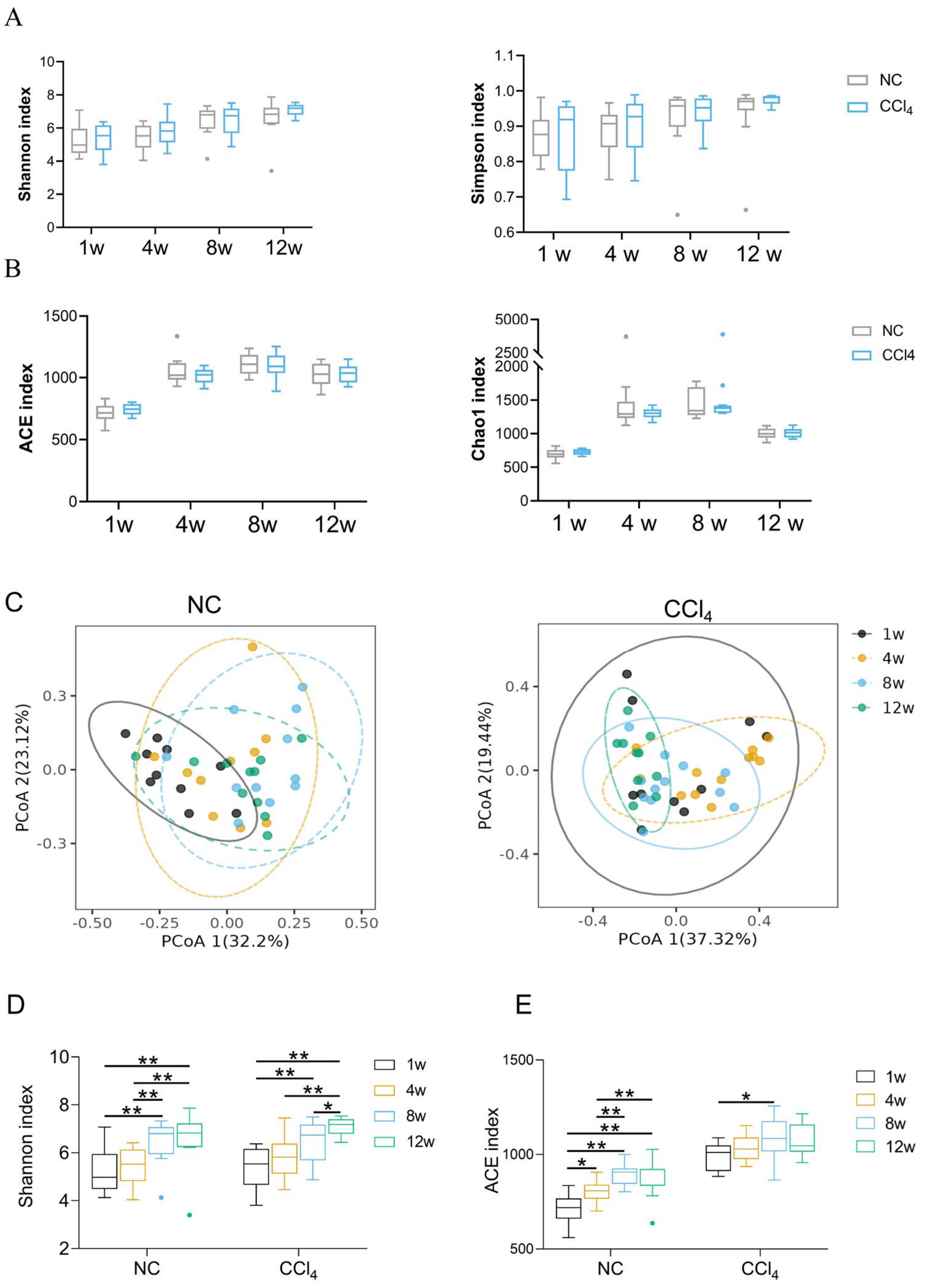

Figure S2.Comparison of α diversity and β diversity. (A) Microbial diversity (Shannon index and Simpson index) and (B) microbial richness (ACE index and Chao1 index) between NC groups and CCl_4 groups. Box plots show the median ± interquartile range (IQR) and 1.5 IQR ranges (whiskers), with outliers denoted by dots. (C) PCoA plots of microbial communities based on weighted_UniFrac distance. (D) Microbial diversity (Shannon index) and (E) microbial richness (ACE index) at different time points (n=10-12).*p<0.05, **P<0.01.

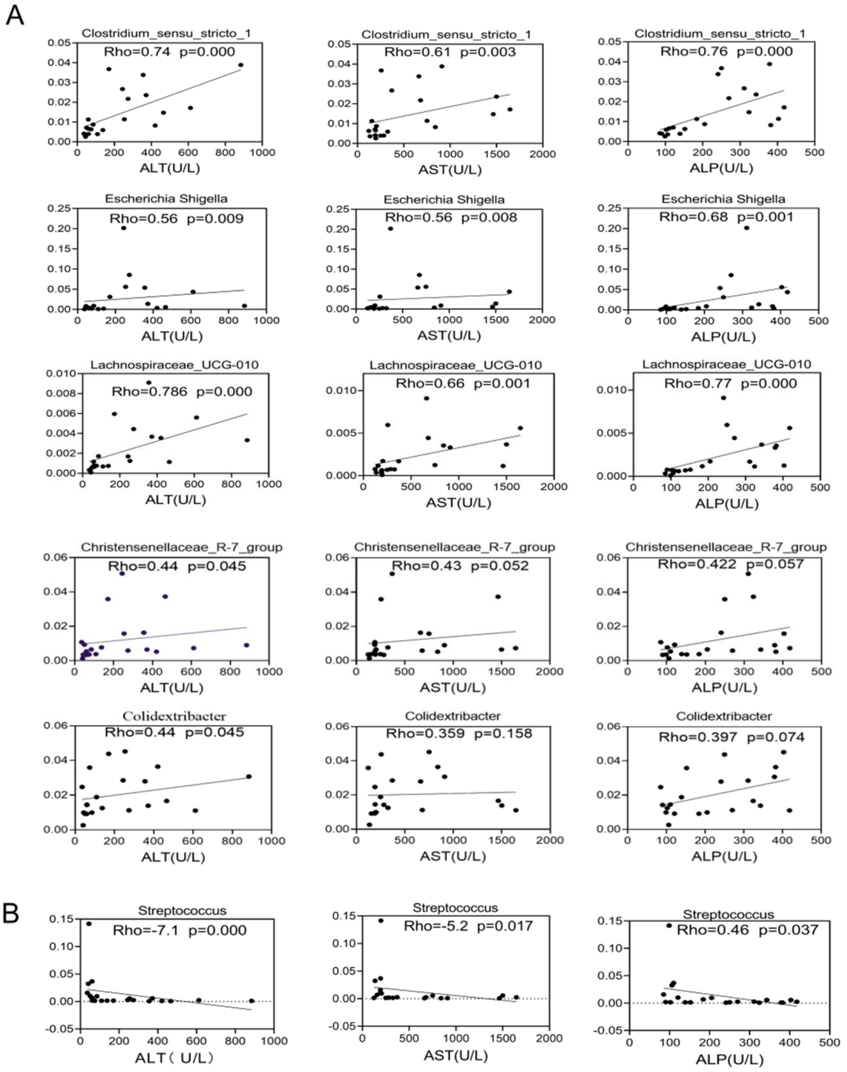

Figure S3. Correlation between distinctive gut microbiota and serum ALT, AST and ALP levels at the advanced fibrosis stage. (A) Positive correlation between distinctive gut microbiota and serum ALT, AST and ALP levels. (B)Negative correlation between distinctive gut microbiota and serum ALT, AST and ALP levels (n=21).

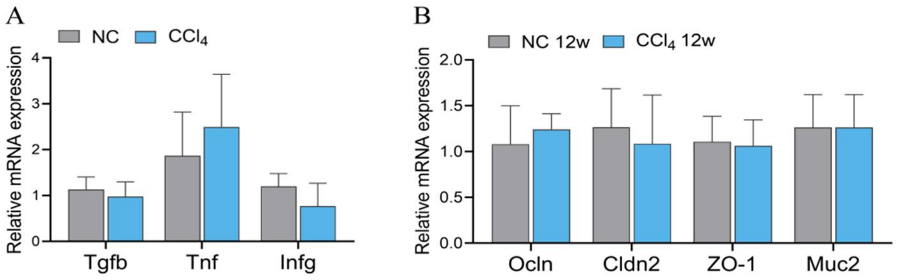

Figure S4. Assessment of the mRNA levels of inflammatory factors and barrier proteins in the advanced fibrosis stage in the colon (n=3-5). Gene expression of inflammatory factors (A) and barrier proteins (B) in the colon. Each value is expressed as the mean ± SEM.

gene name	direction	5' to 3'
Acta2	Forward	AATGACCCAGATTATGTTTGAGACC
	Reverse	ATCTCCAGAGTCCAGCACAATACC
Col1a1	Forward	GTCAGACCTGTGTGTTCCCC
	Reverse	GATCGGAACCTTCGCTTCCA
Ifng	Forward	ACAACCCACAGATCCAGCAC
	Reverse	CCAGAATCAGCACCGACTCC
Tnf	Forward	CATCCGTTCTCTACCCAGCC
	Reverse	AATTCTGAGCCCGGAGTTGG
Tgfb	Forward	CTGCTGACCCCCACTGATAC
	Reverse	TCGACGTTTGGGACTGATCC
Il10	Forward	TTGAACCACCCGGCATCTAC
	Reverse	CCAAGGAGTTGCTCCCGTTA
Il1b	Forward	GACTTCACCATGGAACCCGT
	Reverse	CAGGGAGGGAAACACACGTT
Il6	Forward	TCTGGTCTTCTGGAGTTCCG
	Reverse	AGCATTGGAAGTTGGGGTAGG
Muc2	Forward	TCTGCATTGACTGGCGGAAT
	Reverse	GTCATAGCCAGGGGCAAACT
ZO-1	Forward	AGATGAGCGGGCTACCTTAT
	Reverse	TCATGGGAGCGAACTGAATG
Ocln	Forward	CCCTTCTTTCCTTAGGCGACC
	Reverse	GTGCATCTCTCCGCCATACA
Cldn2	Forward	GACTGTGGATGTCCTGCGTT
	Reverse	CATCAAGGCTCTGGTTGCCT
Cyp7a1	Forward	GAATTGCCGTGTTGGTGAGC
	Reverse	CCCAGGTACGGAATCAACCC
Cyp8b1	Forward	CAGTACACATGGACCCCGAC
	Reverse	AGGGCATGTTGTAGTGGTGG
Cyp27a1	Forward	GTGATTAAGGAGACCCTGCG
	Reverse	CCGGAGTTATCGTCCTCTCT
Cyp7b1	Forward	TTTCTGGGCATTCAGCATCCA
	Reverse	TGTGCAGCCTTATTCCGCTA
Bsep	Forward	TGGGGCTCGTCAGATAAGGA
	Reverse	ACATGCGCTGGAGGAAATGA
Mrp2	Forward	TGCCCATTATCCGTGCCTTT
	Reverse	GAACAAAGCCCACAACGTCC
Asbt	Forward	CAAATGGCCCCAAAAAGCGA
	Reverse	CTGTACCAGGGTTGACCAGC
Oatp1	Forward	TTGCGGGCTGTAAGAAGTTT

	Reverse	ACCCAGGTATGGCTGTAAGT
Ostα	Forward	TCCATCTTGGCTAACAGCGG
	Reverse	TTGAGCGTTGAGTCCAGGTC
Ostβ	Forward	TGGTGATGGTGATAGGCGTG
	Reverse	GCGTCTCTCTTAGGATGCCC
Ntcp	Forward	AAGGGGGACATGAACCTCAG
	Reverse	TTGGACTTGAGGACGATCCC
Mrp3	Forward	AACACTCCAGACCTCACACC
	Reverse	GGACTATGTAGCCAAGCCGA
Mrp4	Forward	TGCAAGCCACATCCTCATAC
	Reverse	AGGGTCTGGATGACTGTTGA
Fxr	Forward	GGGGAGCTGAAAATGACCCA
	Reverse	AAATGCTGAGGGTTCTCGGG
Shp	Forward	GCTAGAGGAACCCAACAGTG
	Reverse	AGTGCCTTTCAGGTATGCGT
Ibabp	Forward	GTCTTACTCTGGGGGCAACA
	Reverse	CACCCTCCATCTTCACGGTT
Fgf15	Forward	AGGAGGACCAGAACGAACGA
	Reverse	GAGTAGCGAATCAGCCCGTA
Gapdh	Forward	AAGAGAGAGGCCCTCAGTTGCCTGA
	Reverse	CCCAGGCCCCTCCTGTTGTTAT

Supplementary Table S1. Primer sequences for RT-PCR.

p value of AMOVA analysis at the same time point of different groups			
NC 1w vs CCl_4 1w	NC 4w vs CCl_4 4w	NC 8w vs CCl_4 8w	NC 12w vs CCl_4 12w
0.001	0.002	0.23	0.02
p value of AMOVA analysis between different time points			
p[a]	NC group		
	1w	4w	8w
4w	0.004		
8w	0.003	0.048	
12w	0.003	0.006	0.048
p[a]	CCl_4 group		
	1w	4w	8w
4w	0.028		
8w	0.016	0.008	
12w	0.016	0.003	0.003

Supplementary Table S2. Difference analysis of β diversity by AMOVA method. a, P values were corrected by the method of Benjamini-Hochberg.

CCl_4 1w vs NC 1w

phylum	class	order	family	genus	species
Actinobacteriota	Actinobacteria	Corynebacteriales	Corynebacteriaceae	Corynebacterium	Corynebacterium_urealyticum
			Dietziaceae	Dietzia	
	Coriobacteriia	Coriobacteriales	Eggerthellaceae	Adlercreutzia	
			Coriobacteriaceae	Collinsella	Collinsella_provencensis
Bacteroidota	Bacteroidia	Bacteroidales	Bacteroidaceae	Bacteroides	
			F082		
			Marinifilaceae	Odoribacter	
			Prevotellaceae	Prevotella	
			Rikenellaceae	Rikenellaceae_RC9_gut_group	
			Tannerellaceae	Parabacteroides	
Campilobacterota	Campylobacteria	Campylobacterales	Helicobacteraceae	Helicobacter	Helicobacter_bilis
Desulfobacterota	Desulfovibrionia	Desulfovibrionales	Desulfovibrionaceae	Desulfovibrio	Desulfovibrio_sp_UNSW3caefatS
Firmicutes	Bacilli	Lactobacillales	Lactobacillaceae	Lactobacillus	Lactobacillus_intestinalis
					Lactobacillus_johnsonii
					Lactobacillus_murinus
					Lactobacillus_reuteri
					Lactobacillus_sp_KC38
			Streptococcaceae	Streptococcus	Streptococcus_respiraculi
			Vagococcaceae	Vagococcus	
		Bacillales	Bacillaceae	Oceanobacillus	
		Erysipelotrichales	Erysipelotrichaceae	Dubosiella	
		Izemoplasmatales			

		Staphylococcales	Gemellaceae	Gemella	
	Clostridia	Christensenellales	Christensenellaceae	unidentified_Christensenellaceae	Christensenella_sp_Marseille_P2437.
		Clostridia_UCG_014			
		Clostridiales	Clostridiaceae	Candidatus_Arthromitus	
		Lachnospirales	Lachnospiraceae	Blautia	Lachnospiraceae_bacterium_28_4
					Lachnospiraceae_bacterium_615
					Lachnospiraceae_bacterium_DW59
				Lachnospira	
				Lachnospiraceae_UCG_001	
		Oscillospirales	Oscillospiraceae	Colidextribacter	
			_Eubacterium__coprostanoligenes_group	unidentified__Eubacterium__coprostanoligenes_group	
				Candidatus_Soleaferrea	Ruminococcaceae_bacterium_GD6
	Negativicutes	Veillonellales_Selenomonadales	Selenomonadaceae	Quinella	
			Veillonellaceae	Veillonella.	
Proteobacteria	Gammaproteobacteria	Burkholderiales	Alcaligenaceae	Oligella	
			Neisseriaceae	Neisseria	Neisseria_sp_10022
		Oceanospirillales	Alcanivoraceae1	Alcanivorax	
		Pseudomonadales	Moraxellaceae	Acinetobacter	Acinetobacter_lwoffii
Spirochaetota	Spirochaetia	Spirochaetales	Spirochaetaceae	Treponema	

unidentified_Bacteria	Clostridia	Oscillospirales	Butyricicoccaceae		
			Oscillospiraceae		
	Saccharimonadia	Saccharimonadales	Saccharimonadaceae	Candidatus_Saccharimonas	
**CCl_4 4w vs NC 4w**					
**phylum**	**class**	**order**	**family**	**genus**	**species**
Actinobacteriota	Actinobacteria	Corynebacteriales	Corynebacteriaceae	Corynebacterium	Corynebacterium_glutamicum.
					Corynebacterium_urealyticum
			Atopobiaceae	Coriobacteriaceae_UCG_002	
			Coriobacteriaceae	Collinsella	Collinsella_provencensis
			Eggerthellaceae	DNF00809	
Bacteroidota	Bacteroidia	Bacteroidales	Rikenellaceae	Alistipes	Alistipes_indistinctus
Desulfobacterota	Desulfovibrionia	Desulfovibrionales	Desulfovibrionaceae	Desulfovibrio	Desulfovibrio_sp_UNSW3caefatS
Fibrobacterota	Fibrobacteria	Fibrobacterales	Fibrobacteraceae	Fibrobacter	Fibrobacter_intestinalis
Firmicutes	Bacilli	Lactobacillales	Lactobacillaceae	Lactobacillus	Lactobacillus_murinus
		Erysipelotrichales	Erysipelatoclostridiaceae	Erysipelatoclostridium	Clostridiales_bacterium_CHKCI006
	Clostridia	Christensenellales	Christensenellaceae	Christensenellaceae_R_7_group	Christensenella_sp_Marseille_P3954
		Clostridiales	Clostridiaceae	Clostridium_sensu_stricto_1	Clostridium_sp_ND2
		Lachnospirales	Lachnospiraceae	Roseburia	
				Lachnospiraceae_ND3007_group	
				Lachnospiraceae_UCG_010	
				Coprococcus	

		Oscillospirales	Ruminococcaceae	Ruminococcus	Ruminococcus_sp_N15_MGS_57
				Caproiciproducens	
				Anaerotruncus	
			Oscillospiraceae	Colidextribacter	
		Peptostreptococcales_Tissierellales	Anaerovoracaceae	_Eubacterium__nodatum_group	
	Negativicutes	Veillonellales_Selenomonadales	Veillonellaceae		
	unidentified_Firmicutes	DTU014			
Proteobacteria	Gammaproteobacteria	Burkholderiales	Neisseriaceae	Neisseria	Neisseria_sp_10022
			Sutterellaceae	Parasutterella	Burkholderiales_bacterium_YL45
Spirochaetota	Spirochaetia	Spirochaetales	Spirochaetaceae	Treponema	
Verrucomicrobiota	Verrucomicrobiae	Verrucomicrobiales	Akkermansiaceae	Akkermansia	Akkermansia_muciniphila
**CCl_4 8w vs NC 8w**					
**phylum**	**class**	**order**	**family**	**genus**	**species**
Bacteroidota	Bacteroidia	Bacteroidales	Rikenellaceae	Rikenellaceae_RC9_gut_group	
			Bacteroidaceae	Bacteroides	Bacteroides_caecigallinarum
Firmicutes	Bacilli	Lactobacillales	Enterococcaceae	Enterococcus	
			Streptococcaceae	Streptococcus	Streptococcus_hyointestintinalis
			Lactobacillaceae	Lactobacillus	Lactobacillus_murinus

	Clostridia	Clostridiales	Clostridiaceae	Clostridium_sensu_stricto_1	
		Lachnospirales	Lachnospiraceae	Lachnospiraceae_UCG_010	
		Oscillospirales	Ruminococcaceae	Ruminococcus	Ruminococcaceae_bacterium_Marseille_3738
unidentified_Bacteria	Saccharimonadia	Saccharimonadales	Saccharimonadaceae	Candidatus_Saccharimonas	

CCl_4 12w vs NC 12w

phylum	class	order	family	genus	species
Actinobacteriota	Coriobacteriia	Coriobacteriales	Eggerthellaceae	Adlercreutzia	
				Parvibacter	
Bacteroidota	Bacteroidia	Bacteroidales	Bacteroidaceae	Bacteroides	Bacteroides_sartorii
			Barnesiellaceae	Barnesiella	
			Prevotellaceae	Prevotellaceae_UCG_003	
				Prevotellaceae_UCG_004	
			Rikenellaceae	Rikenellaceae_RC9_gut_group	
Desulfobacterota	Desulfovibrionia	Desulfovibrionales	Desulfovibrionaceae	Bilophila	
				Desulfovibrio	Desulfovibrio_fairfieldensi
Firmicutes	Bacilli	Bacillales	Planococcaceae	Kurthia	Kurthia_sp_11kri321
		Erysipelotrichales	Erysipelotrichaceae	Turicibacter	
		Lactobacillales	Enterococcaceae	Enterococcus	
			Lactobacillaceae	Lactobacillus	Lactobacillus_murinus
			Streptococcaceae	Streptococcus	Streptococcus_hyointestinalis
	Clostridia	Christensenellales	Christensenellaceae	Christensenella	
				Christensenellaceae_R_7_group	

		Clostridiales	Clostridiaceae	Clostridium_sensu_stricto_1	
		Lachnospirales	Lachnospiraceae	Anaerostipes	
				A2	
				Lachnospiraceae_UCG_010	
		Oscillospirales	Oscillospiraceae	Colidextribacter	
			_Clostridium__methylpentosum_group		
			_Eubacterium__coprostanoligenes_group	unidentified__Eubacterium__coprostanoligenes_group	
			Ruminococcaceae	Paludicola	
	Negativicutes	Acidaminococcales	Acidaminococcaceae	Phascolarctobacterium	
		Veillonellales_Selenomonadales	Selenomonadaceae		
Proteobacteria	Gammaproteobacteria	Enterobacterales	Enterobacteriaceae	Escherichia_Shigella	Escherichia_coli
		Burkholderiales	Sutterellaceae		
unidentified_Bacteria	Clostridia	Oscillospirales	Ruminococcaceae	unidentified_Ruminococcaceae	_Clostridium__leptum
Verrucomicrobiota	Verrucomicrobiae	Verrucomicrobiales	Akkermansiaceae	Akkermansia	

Supplementary Table 3. Differences in the gut microbiota at different fibrosis stages.

LDA>3, red color represents taxa increased in CCl_4 group, green color represents taxa decreased in CCl_4 group, and black color represents no difference between the two groups.

Phylum	NC group							
	Week 1		Week 4		Week 8		Week 12	
	mean[a]%	SD[b]%	mean[a]%	SD[b]%	mean[a]%	SD[b]%	mean[a]%	SD[b]%
Firmicutes	57.43	12.87	54.23	15.56	45.05	13.04	52.93	15.38
Bacteroidota	27.56	13.80	30.16	15.73	28.69	10.32	29.56	12.69
Spirochaetota	1.01	1.77	8.82	14.25	11.90	11.86	4.00	5.55
Proteobacteria	10.15	14.40	0.79	0.49	1.05	0.60	1.93	1.51
Actinobacteriota	0.63	0.22	0.43	0.11	1.48	3.39	0.76	0.60
Verrucomicrobiota	0.09	0.05	0.07	0.04	1.84	3.12	1.82	3.41
unidentified_Bacteria	1.27	0.71	2.71	2.17	4.06	1.58	4.16	1.75
Fibrobacterota	0.19	0.55	0.24	0.27	0.82	0.69	0.28	0.14
Campilobacterota	0.35	0.36	0.24	0.09	0.56	0.46	0.32	0.52
Desulfobacterota	0.16	0.12	0.15	0.17	0.12	0.09	0.38	0.33
Cyanobacteria	0.04	0.05	0.02	0.01	0.16	0.31	0.04	0.03
Deferribacteres	0.04	0.06	0.03	0.02	0.04	0.04	0.01	0.01
Fusobacteriota	<0.01	<0.01	<0.01	<0.01	0.03	0.03	0.01	0.02

Phylum	CCl_4 group							
	Week 1		Week 4		Week 8		Week 12	
	mean[a]%	SD[b]%	mean[a]%	SD[b]%	mean[a]%	SD[b]%	mean[a]%	SD[b]%
Firmicutes	28.04	6.83	31.56	13.64	36.53	13.19	47.39	9.85
Bacteroidota	38.54	19.09	30.27	9.02	30.28	9.53	30.12	12.10
Proteobacteria	12.18	19.62	1.71	1.23	1.45	0.94	8.12	7.12
Spirochaetota	14.22	16.25	25.07	16.58	10.83	10.01	3.50	3.50
Verrucomicrobiota	0.01	0.01	3.81	5.95	5.41	6.80	0.05	0.03
Fibrobacterota	0.02	0.01	0.04	0.03	1.43	2.74	0.48	0.43
unidentified_Bacteria	4.15	2.42	2.36	0.69	2.82	1.21	3.51	1.02
Actinobacteriota	0.70	0.36	0.35	0.19	0.91	1.59	1.33	0.75
Fusobacteriota	0.04	0.04	0.00	0.00	0.37	1.15	0.03	0.04
Desulfobacterota	0.46	0.46	0.23	0.15	0.10	0.03	0.27	0.16
Campilobacterota	0.43	0.32	0.37	0.22	0.46	0.34	0.38	0.34
Deferribacteres	0.11	0.12	0.03	0.02	0.03	0.03	0.07	0.10
Cyanobacteria	0.05	0.04	0.03	0.03	0.06	0.05	0.01	0.01
Elusimicrobia	<0.01	<0.01	<0.01	<0.01	0.01	0.02	<0.01	<0.01

Supplementary Table S4. The relative abundance of major phyla at different ages (average

relative abundance > 0.01 %). mean[a], average relative abundance; SD[b], standard deviation.

genus	NC group							
	Week 1		Week 4		Week 8		Week 12	
	mean[a] %	SD[b] %	mean[a] %	SD[b] %	mean[a] %	SD[b] %	mean[a] %	SD[b] %
Lactobacillus	38.92	18.16	24.30	15.48	15.42	17.64	16.50	21.02
Treponema	1.00	1.77	8.80	14.25	11.88	11.87	3.99	5.54
Prevotellaceae_UCG-003	2.60	2.33	10.22	13.34	2.54	2.58	4.58	6.41
Ruminococcus	1.09	0.78	12.53	6.42	3.59	1.16	2.13	1.77
Escherichia-Shigella	9.55	14.48	0.37	0.36	0.20	0.10	0.36	0.28
Bacteroides	1.57	1.11	2.45	1.64	3.27	1.55	1.96	1.19
Romboutsia	3.05	4.32	0.96	0.31	0.79	0.34	1.75	0.99
Lachnospiraceae_NK4A136_group	0.47	0.47	0.85	0.64	3.08	3.70	1.92	1.75
Rikenellaceae_RC9_gut_group	1.30	0.52	2.26	1.67	1.48	0.48	0.83	0.41
Candidatus_Saccharimonas	0.52	0.44	1.57	1.81	1.89	1.13	1.73	1.49
Prevotella	0.35	0.65	0.46	0.33	2.53	2.47	1.46	2.51
Prevotellaceae_UCG-001	0.68	0.59	1.64	0.98	1.88	3.32	0.25	0.22
NK4A214_group	0.24	0.10	1.11	0.87	1.05	0.48	1.53	1.09
unidentified_[Eubacterium]_coprostanoligenes_group	2.30	6.14	0.97	1.19	0.43	0.39	0.17	0.15
Akkermansia	0.09	0.05	0.07	0.04	1.84	3.12	1.82	3.41
Streptococcus	0.17	0.39	0.26	0.19	0.84	1.39	2.36	4.10
Colidextribacter	0.26	0.12	0.43	0.19	1.15	0.48	1.47	0.91
Turicibacter	1.31	1.43	0.39	0.15	0.40	0.25	0.56	0.37
Parabacteroides	0.42	0.19	0.67	0.39	0.72	0.25	0.64	0.29
[Eubacterium]_xylanophilum_group	0.34	0.55	0.30	0.21	0.74	0.57	0.96	0.64
Monoglobus	0.21	0.11	0.32	0.15	0.79	0.69	0.96	0.53
Dubosiella	0.70	0.48	0.19	0.15	0.63	1.05	0.51	0.69
Christensenellaceae_R-7_group	0.30	0.28	0.66	0.52	0.52	0.48	0.54	0.29
Alistipes	0.58	0.54	0.36	0.27	0.21	0.10	0.75	1.29
Parasutterella	0.50	0.32	0.22	0.07	0.59	0.57	0.37	0.48
Alloprevotella	0.25	0.19	0.42	0.22	0.82	0.75	0.11	0.09
UCG-005	0.17	0.12	0.50	0.27	0.47	0.23	0.42	0.39
Fibrobacter	0.19	0.55	0.24	0.27	0.82	0.69	0.28	0.14
Allobaculum	0.10	0.05	0.08	0.03	0.52	1.01	0.83	1.71
Clostridium_sensu_stricto_1	0.27	0.13	0.35	0.13	0.28	0.10	0.58	0.26

Roseburia	0.11	0.09	0.13	0.08	0.58	0.48	0.67	0.59
Helicobacter	0.35	0.36	0.24	0.09	0.56	0.45	0.31	0.52
Bifidobacterium	0.10	0.09	0.07	0.04	1.11	3.30	0.13	0.22
Anaerostipes	<0.01	<0.01	0.01	0.02	0.03	0.03	1.26	1.51
Prevotellaceae_NK3B31_group	0.02	0.04	0.08	0.08	0.90	2.05	0.05	0.06
Enterococcus	0.33	0.38	0.52	1.04	0.08	0.07	0.10	0.07
Barnesiella	0.52	0.79	0.40	0.70	0.07	0.04	0.03	0.03
Odoribacter	0.58	0.58	0.08	0.06	0.14	0.13	0.08	0.05
Papillibacter	0.13	0.11	0.15	0.09	0.26	0.21	0.29	0.17
Desulfovibrio	0.16	0.12	0.15	0.16	0.11	0.09	0.37	0.33
Quinella	0.03	0.03	0.16	0.21	0.15	0.08	0.32	0.25
Intestinimonas	0.06	0.05	0.12	0.05	0.20	0.09	0.23	0.16
Vagococcus	0.55	0.66	0.03	0.02	0.02	0.01	0.02	<0.01
Corynebacterium	0.20	0.20	0.06	0.04	0.10	0.07	0.19	0.21
Anaerobiospirillum	0.03	0.02	0.11	0.15	0.07	0.07	0.18	0.29
Enterorhabdus	0.12	0.09	0.09	0.04	0.09	0.06	0.07	0.04
Lachnospiraceae_UCG-010	0.08	0.03	0.15	0.09	0.07	0.03	0.07	0.04
Oscillibacter	0.03	0.02	0.06	0.02	0.14	0.12	0.14	0.11
Dorea	0.03	0.04	0.14	0.17	0.11	0.15	0.08	0.11
Rodentibacter	0.01	0.02	0.02	0.01	0.06	0.05	0.26	0.26
Adlercreutzia	0.12	0.04	0.10	0.04	0.05	0.02	0.08	0.04
Blautia	0.20	0.54	0.03	0.06	0.05	0.04	0.05	0.03
[Eubacterium]_ruminantium_group	0.01	0.01	0.02	<0.01	0.24	0.19	0.03	0.02
[Ruminococcus]_torques_group	0.04	0.02	0.05	0.04	0.09	0.03	0.08	0.03
[Eubacterium]_siraeum_group	0.03	0.02	0.04	0.05	0.13	0.25	0.03	0.03
unidentified_Muribaculaceae	0.07	0.09	0.03	0.02	0.05	0.04	0.09	0.05
Lachnospiraceae_UCG-006	<0.01	<0.01	0.02	0.02	0.05	0.04	0.13	0.12
Sutterella	0.03	0.05	0.02	0.02	0.09	0.05	0.05	0.06
Frisingicoccus	0.06	0.10	0.03	0.03	0.06	0.04	0.03	0.02
Neisseria	<0.01	<0.01	<0.01	<0.01	<0.01	<0.01	0.17	0.24
UCG-009	0.01	0.01	0.04	0.03	0.05	0.03	0.06	0.03
Jeotgalicoccus	0.02	0.01	0.02	<0.01	0.03	0.02	0.10	0.13
Family_XIII_AD3011_group	0.02	0.01	0.02	0.01	0.06	0.04	0.06	0.06
Prevotellaceae_Ga6A1_group	<0.01	<0.01	0.04	0.09	0.03	0.02	0.08	0.12
Butyricimonas	0.05	0.04	0.04	0.03	0.04	0.02	0.02	0.01
Acinetobacter	<0.01	<0.01	<0.01	<0.01	<0.01	<0.01	0.12	0.18
Phascolarctobacterium	0.02	0.02	0.02	0.02	0.05	0.02	0.03	0.05
Lachnoclostridium	0.02	0.01	0.02	0.01	0.04	0.02	0.05	0.05
Mucispirillum	0.04	0.06	0.03	0.02	0.04	0.04	0.01	0.01
Lachnospira	0.02	0.02	0.02	<0.01	0.04	0.03	0.03	0.03
Anaerovorax	0.03	0.02	0.02	0.01	0.04	0.03	0.02	0.01
Anaerovibrio	0.07	0.08	<0.01	<0.01	<0.01	<0.01	0.03	0.06
Rothia	<0.01	<0.01	<0.01	<0.01	<0.01	<0.01	0.09	0.19

Prevotellaceae_UCG-004	<0.01	<0.01	0.01	<0.01	0.06	0.08	0.02	0.02
Eubacterium	0.03	0.06	0.02	0.02	0.02	0.01	0.02	0.02
Tuzzerella	0.02	0.02	0.02	0.01	0.03	0.02	0.03	0.02
GCA-900066575	0.02	0.02	0.03	0.02	0.02	0.02	0.02	<0.01
Negativibacillus	0.01	0.01	0.03	0.04	0.03	0.02	0.02	0.03
Marvinbryantia	0.02	0.02	0.03	0.01	0.02	<0.01	0.02	0.02
unidentified_Clostridia_vadinBB60_group	0.01	<0.01	0.03	0.02	0.02	0.02	0.02	0.01
Anaeroplasma	0.06	0.17	<0.01	0.01	0.01	0.01	<0.01	<0.01
Faecalibaculum	<0.01	<0.01	<0.01	<0.01	0.05	0.08	0.02	0.03
Lachnospiraceae_NK4B4_group	<0.01	<0.01	<0.01	<0.01	0.04	0.03	0.03	0.02
Erysipelatoclostridium	0.04	0.07	0.01	0.01	0.02	0.01	<0.01	<0.01
Mycoplasma	0.03	0.04	0.02	0.02	0.02	0.03	<0.01	<0.01
Rikenella	0.02	0.02	0.02	0.05	0.02	0.05	<0.01	0.01
unidentified_Christensenellaceae	0.01	<0.01	0.02	0.01	0.01	<0.01	0.03	0.01
dgA-11_gut_group	0.02	0.01	0.05	0.08	<0.01	<0.01	<0.01	<0.01
Staphylococcus	0.02	0.02	<0.01	<0.01	0.01	<0.01	0.03	0.03
unidentified_Ruminococcaceae	<0.01	0.01	0.02	0.01	0.03	0.02	0.01	0.01
Veillonella	<0.01	<0.01	<0.01	<0.01	0.01	<0.01	0.04	0.04
Defluviitaleaceae_UCG-011	0.01	<0.01	0.02	0.02	0.01	<0.01	0.01	0.01
Coprococcus	<0.01	<0.01	<0.01	<0.01	0.03	0.02	0.03	0.02
Sporosarcina	0.01	0.01	<0.01	<0.01	0.01	0.01	0.04	0.05
Collinsella	0.01	0.02	0.02	0.02	0.01	<0.01	0.01	<0.01
Paenalcaligenes	<0.01	<0.01	<0.01	<0.01	0.01	<0.01	0.04	0.06
Pygmaiobacter	<0.01	<0.01	<0.01	<0.01	0.01	<0.01	0.03	0.03
Erysipelotrichaceae_UCG-003	<0.01	0.02	0.01	0.02	<0.01	<0.01	0.02	0.04
[Eubacterium]_nodatum_group	<0.01	<0.01	<0.01	0.01	0.02	0.01	0.02	<0.01
DNF00809	<0.01	<0.01	0.02	0.01	<0.01	<0.01	0.01	0.01
Coriobacteriaceae_UCG-002	<0.01	<0.01	<0.01	<0.01	0.03	0.07	<0.01	0.01
[Clostridium]_innocuum_group	0.02	0.06	<0.01	<0.01	0.02	0.02	<0.01	0.01
UBA1819	0.01	0.01	<0.01	<0.01	0.01	<0.01	0.02	0.01
Atopostipes	<0.01	<0.01	<0.01	<0.01	<0.01	<0.01	0.04	0.06
Fusobacterium	<0.01	<0.01	<0.01	<0.01	0.03	0.03	0.01	0.02
unidentified_Xanthomonadaceae	<0.01	<0.01	0.01	0.01	<0.01	<0.01	0.02	0.04
Facklamia	<0.01	<0.01	<0.01	<0.01	<0.01	<0.01	0.03	0.03
Oligella	<0.01	<0.01	<0.01	<0.01	<0.01	<0.01	0.02	0.03
Ileibacterium	<0.01	<0.01	<0.01	<0.01	0.04	0.09	<0.01	<0.01
UCG-004	<0.01	0.01	0.02	0.03	<0.01	<0.01	0.01	0.03
Granulicatella	<0.01	<0.01	<0.01	<0.01	<0.01	<0.01	0.04	0.07
Butyricicoccus	<0.01	<0.01	<0.01	<0.01	0.01	<0.01	0.01	<0.01
Globicatella	<0.01	<0.01	<0.01	<0.01	<0.01	<0.01	0.03	0.05
Pseudomonas	<0.01	<0.01	<0.01	<0.01	<0.01	<0.01	0.02	0.02
Harryflintia	<0.01	<0.01	<0.01	<0.01	<0.01	0.01	0.01	0.01

Brachybacterium	<0.01	<0.01	<0.01	<0.01	<0.01	<0.01	0.02	0.04
Psychrobacter	<0.01	<0.01	<0.01	<0.01	<0.01	<0.01	0.02	0.02
Epulopiscium	<0.01	<0.01	<0.01	<0.01	<0.01	<0.01	0.03	0.02
Pseudogracilibacillus	<0.01	<0.01	<0.01	<0.01	<0.01	<0.01	0.03	0.06
Gemella	<0.01	<0.01	<0.01	<0.01	<0.01	<0.01	0.03	0.06
Acetatifactor	0.01	0.03	<0.01	<0.01	0.01	0.01	<0.01	<0.01
[Eubacterium]_brachy_group	<0.01	<0.01	<0.01	<0.01	<0.01	<0.01	0.01	<0.01
Anaerofilum	<0.01	<0.01	<0.01	<0.01	<0.01	<0.01	0.01	0.01
Aerococcus	0.01	<0.01	<0.01	<0.01	<0.01	<0.01	<0.01	<0.01
Dietzia	<0.01	<0.01	<0.01	<0.01	<0.01	0.01	0.01	0.01
Ruminiclostridium	<0.01	<0.01	<0.01	<0.01	0.01	0.01	<0.01	<0.01
Muribaculum	<0.01	<0.01	<0.01	<0.01	0.02	0.02	<0.01	<0.01
Paraprevotella	<0.01	<0.01	<0.01	<0.01	0.02	0.02	<0.01	<0.01
Brevibacterium	<0.01	<0.01	<0.01	<0.01	<0.01	<0.01	0.01	0.02
Coprobacillus	<0.01	0.01	<0.01	<0.01	0.01	0.01	<0.01	<0.01
Clostridium_sensu_stricto_13	<0.01	<0.01	<0.01	<0.01	<0.01	<0.01	0.01	0.01
Citrobacter	<0.01	<0.01	<0.01	<0.01	<0.01	<0.01	0.01	0.03
Actinomyces	<0.01	<0.01	<0.01	<0.01	<0.01	<0.01	0.01	0.03
Lautropia	<0.01	<0.01	<0.01	<0.01	<0.01	<0.01	0.01	0.02
**genus**	**CCl_4 group**							
	**Week 1**		**Week 4**		**Week 8**		**Week 12**	
	mean[a]%	SD[b]%	mean[a]%	SD[b]%	mean[a]%	SD[b]%	mean[a]%	SD[b]%
Treponema	14.22	17.12	25.05	16.58	10.81	10.02	3.48	3.50
Lactobacillus	4.76	4.46	4.39	4.15	8.47	11.38	8.25	4.87
Escherichia-Shigella	11.59	20.52	0.81	0.96	0.32	0.37	5.04	5.94
Rikenellaceae_RC9_gut_group	3.28	2.08	3.40	1.62	3.09	2.05	3.62	2.59
Bacteroides	4.20	2.56	2.28	1.15	3.41	3.65	1.39	0.74
Prevotellaceae_UCG-003	3.75	3.57	4.73	2.93	1.43	0.55	1.25	1.97
Akkermansia	<0.01	0.01	3.81	5.95	5.40	6.81	0.03	0.03
Ruminococcus	1.01	0.77	3.64	3.28	2.45	1.07	1.89	1.44
Lachnospiraceae_NK4A136_group	0.57	0.49	1.25	0.71	2.46	1.53	2.00	1.21
Colidextribacter	0.88	0.57	1.05	0.66	1.35	0.39	2.64	1.29
Prevotellaceae_UCG-001	0.70	0.73	3.29	2.96	1.56	1.17	0.09	0.09
Romboutsia	2.65	1.95	1.11	0.35	0.63	0.24	1.03	0.44
Candidatus_Saccharimonas	2.27	2.45	0.99	0.45	1.00	0.81	0.78	0.55
Christensenellaceae_R-7_group	0.53	0.43	1.51	0.99	0.62	0.21	1.90	1.63
Alloprevotella	3.22	8.69	0.71	0.98	0.43	0.45	0.18	0.19
unidentified_[Eubacterium]_coprostanoligenes_group	0.75	0.49	0.93	0.83	0.95	0.88	1.62	1.83
Clostridium_sensu_stricto_1	0.25	0.23	0.54	0.33	0.77	0.78	2.33	1.07
NK4A214_group	0.31	0.20	0.88	0.41	1.00	0.53	1.63	1.03
Parabacteroides	1.39	1.31	0.73	0.26	0.75	0.41	0.66	0.38
Parasutterella	0.38	0.33	0.72	0.71	0.86	0.64	1.42	2.10

Turicibacter	2.38	3.24	0.36	0.09	0.22	0.11	0.18	0.09
Dubosiella	0.25	0.28	0.29	0.32	0.62	0.68	1.82	2.80
Barnesiella	0.87	1.93	0.73	0.98	0.27	0.27	1.02	1.41
Monoglobus	0.27	0.07	0.44	0.25	0.61	0.27	0.83	0.42
[Eubacterium]_xylanophilum_group	0.12	0.09	0.51	0.39	0.85	0.50	0.65	0.49
Enterococcus	0.09	0.06	0.07	0.05	0.31	0.29	1.49	1.72
Fibrobacter	0.02	0.01	0.04	0.03	1.43	2.74	0.47	0.43
UCG-005	0.33	0.34	0.45	0.14	0.60	0.36	0.55	0.27
Prevotella	0.10	0.03	0.27	0.21	0.85	0.70	0.41	0.35
Helicobacter	0.43	0.33	0.37	0.22	0.46	0.34	0.38	0.34
Roseburia	0.16	0.15	0.42	0.31	0.32	0.22	0.49	0.58
Alistipes	0.33	0.14	0.24	0.07	0.25	0.08	0.49	0.37
Lachnospiraceae_UCG-010	0.06	0.03	0.40	0.29	0.33	0.42	0.40	0.25
Desulfovibrio	0.46	0.48	0.23	0.14	0.09	0.03	0.26	0.16
Allobaculum	0.08	0.07	0.12	0.19	0.47	0.70	0.19	0.19
Bifidobacterium	0.02	0.03	0.07	0.10	0.57	1.57	0.18	0.20
Adlercreutzia	0.28	0.30	0.09	0.04	0.08	0.03	0.37	0.19
Papillibacter	0.22	0.34	0.12	0.05	0.11	0.05	0.34	0.24
Quinella	0.24	0.40	0.06	0.05	0.09	0.04	0.26	0.46
Oscillibacter	0.03	0.02	0.08	0.10	0.18	0.11	0.25	0.13
[Ruminococcus]_torques_group	0.04	0.02	0.19	0.32	0.13	0.06	0.16	0.08
Enterorhabdus	0.19	0.10	0.08	0.04	0.07	0.03	0.17	0.11
Corynebacterium	0.11	0.06	0.05	0.03	0.07	0.04	0.28	0.26
Streptococcus	0.04	0.04	0.07	0.04	0.15	0.09	0.24	0.19
Intestinimonas	0.12	0.07	0.13	0.10	0.10	0.04	0.13	0.09
Rodentibacter	0.05	0.04	0.01	<0.01	0.04	0.02	0.37	0.32
Phascolarctobacterium	0.06	0.10	0.04	0.03	0.07	0.04	0.29	0.40
Frisingicoccus	0.07	0.09	0.17	0.14	0.07	0.03	0.14	0.07
Fusobacterium	0.04	0.04	<0.01	<0.01	0.37	1.15	0.03	0.04
Sutterella	0.01	0.01	0.07	0.09	0.10	0.12	0.22	0.24
unidentified_Muribaculaceae	0.01	<0.01	0.03	0.02	0.04	0.03	0.28	0.51
[Eubacterium]_ruminantium_group	<0.01	<0.01	0.04	0.04	0.24	0.18	0.06	0.06
Anaerovibrio	0.29	0.66	0.02	0.02	0.01	0.03	<0.01	<0.01
Prevotellaceae_NK3B31_group	0.05	0.03	0.05	0.04	0.17	0.11	0.03	0.03
Blautia	0.11	0.08	0.05	0.05	0.07	0.08	0.08	0.04
Odoribacter	0.16	0.21	0.05	0.04	0.05	0.03	0.03	0.03
Anaerostipes	<0.01	<0.01	<0.01	<0.01	0.01	0.01	0.26	0.30
dgA-11_gut_group	0.08	0.15	0.17	0.15	<0.01	<0.01	<0.01	0.02
Anaerobiospirillum	0.09	0.14	0.03	0.02	0.03	0.02	0.12	0.18
Mucispirillum	0.11	0.12	0.03	0.02	0.03	0.03	0.08	0.10
Lachnospira	<0.01	<0.01	0.03	0.03	0.11	0.14	0.11	0.20
Family_XIII_AD3011_group	<0.01	<0.01	0.04	0.03	0.12	0.19	0.08	0.08
UCG-009	0.02	0.01	0.06	0.05	0.06	0.03	0.10	0.06

Vagococcus	0.03	0.02	0.03	0.03	0.04	0.05	0.14	0.14
Coprococcus	<0.01	<0.01	<0.01	<0.01	0.06	0.03	0.17	0.12
Butyricimonas	0.09	0.05	0.04	0.03	0.06	0.07	0.04	0.04
Dorea	0.05	0.06	0.08	0.08	0.06	0.04	0.03	0.02
[Eubacterium]_siraeum_group	0.13	0.22	0.04	0.05	0.05	0.03	0.01	0.02
Acinetobacter	<0.01	<0.01	0.02	0.03	<0.01	<0.01	0.16	0.17
Neisseria	<0.01	<0.01	<0.01	<0.01	<0.01	<0.01	0.17	0.20
Staphylococcus	0.02	0.01	0.01	<0.01	0.01	<0.01	0.12	0.12
Lachnoclostridium	0.02	0.02	0.02	0.01	0.05	0.06	0.05	0.04
Jeotgalicoccus	0.03	0.03	0.02	<0.01	0.02	0.02	0.07	0.05
Eubacterium	0.02	<0.01	0.03	0.03	0.02	0.03	0.06	0.04
Negativibacillus	0.02	0.02	0.05	0.07	0.02	0.01	0.03	0.04
Defluviitaleaceae_UCG-011	0.02	0.02	0.05	0.04	0.03	0.02	0.02	0.02
Epulopiscium	<0.01	<0.01	<0.01	<0.01	<0.01	<0.01	0.12	0.11
Lachnospiraceae_NK4B4_group	<0.01	0.01	0.03	0.02	0.06	0.06	0.02	<0.01
Marvinbryantia	0.02	0.02	0.05	0.10	0.02	0.02	0.03	0.03
Prevotellaceae_Ga6A1_group	<0.01	<0.01	0.01	0.01	0.03	0.03	0.07	0.09
unidentified_Christensenellaceae	0.05	0.07	0.03	0.02	<0.01	<0.01	0.02	0.02
Oligella	<0.01	<0.01	0.01	0.01	0.04	0.05	0.05	0.05
Dietzia	<0.01	<0.01	<0.01	<0.01	0.02	0.03	0.08	0.11
Anaerovorax	<0.01	<0.01	0.03	0.02	0.03	0.02	0.03	0.02
Erysipelatoclostridium	0.03	0.07	<0.01	0.01	0.05	0.08	0.01	0.01
GCA-900066575	0.03	0.03	0.02	0.02	0.04	0.02	0.02	0.01
[Clostridium]_innocuum_group	<0.01	<0.01	<0.01	<0.01	0.04	0.07	0.05	0.13
Sporosarcina	0.02	0.01	<0.01	<0.01	0.02	0.01	0.07	0.07
Collinsella	0.03	0.04	<0.01	<0.01	0.02	0.02	0.04	0.06
Paenalcaligenes	<0.01	<0.01	0.01	<0.01	0.02	0.03	0.05	0.05
Rothia	<0.01	<0.01	<0.01	<0.01	<0.01	<0.01	0.07	0.12
Psychrobacter	<0.01	<0.01	<0.01	<0.01	0.02	0.04	0.05	0.04
[Eubacterium]_hallii_group	<0.01	<0.01	<0.01	<0.01	<0.01	<0.01	0.07	0.10
[Eubacterium]_nodatum_group	<0.01	<0.01	0.02	<0.01	0.03	0.03	0.02	0.02
Veillonella	0.02	0.01	<0.01	<0.01	0.02	0.02	0.02	0.02
Butyricicoccus	<0.01	<0.01	<0.01	<0.01	0.02	0.02	0.04	0.02
Coprobacillus	<0.01	<0.01	<0.01	<0.01	0.06	0.18	<0.01	<0.01
Prevotellaceae_UCG-004	0.01	0.02	0.01	<0.01	0.03	0.03	<0.01	0.01
Lachnospiraceae_UCG-006	<0.01	<0.01	<0.01	<0.01	0.03	0.03	0.02	0.01
Tuzzerella	<0.01	<0.01	0.02	0.01	0.02	0.02	0.02	0.01
Faecalibaculum	<0.01	<0.01	<0.01	<0.01	0.03	0.04	0.03	0.03
unidentified_Clostridia_vadinBB60_group	<0.01	<0.01	0.02	0.01	0.02	<0.01	0.01	0.01
Mycoplasma	0.03	0.03	0.02	0.02	0.01	0.02	<0.01	<0.01
Coriobacteriaceae_UCG-002	<0.01	0.01	<0.01	<0.01	0.04	0.08	<0.01	0.01
unidentified_Xanthomonadaceae	<0.01	<0.01	0.02	0.03	0.01	0.03	0.01	0.02

Erysipelotrichaceae_UCG-003	0.01	<0.01	<0.01	<0.01	<0.01	<0.01	0.03	0.03
Candidatus_Arthromitus	0.04	0.05	<0.01	<0.01	<0.01	<0.01	<0.01	<0.01
unidentified_Ruminococcaceae	0.01	0.01	0.01	0.01	0.02	0.02	<0.01	0.01
Ruminiclostridium	<0.01	<0.01	<0.01	<0.01	0.01	<0.01	0.03	0.01
Ileibacterium	<0.01	<0.01	<0.01	<0.01	0.04	0.11	<0.01	<0.01
UBA1819	0.02	<0.01	<0.01	<0.01	<0.01	<0.01	0.01	0.02
Paludicola	<0.01	<0.01	0.01	<0.01	<0.01	<0.01	0.02	<0.01
Facklamia	<0.01	<0.01	<0.01	<0.01	<0.01	<0.01	0.02	0.01
DNF00809	<0.01	<0.01	<0.01	0.01	<0.01	<0.01	0.01	0.01
Sphaerochaeta	<0.01	<0.01	<0.01	<0.01	<0.01	0.01	0.01	0.02
Gemella	<0.01	<0.01	<0.01	<0.01	<0.01	<0.01	0.03	0.05
Granulicatella	<0.01	<0.01	<0.01	<0.01	<0.01	<0.01	0.03	0.06
Pygmaiobacter	<0.01	<0.01	0.01	0.01	<0.01	<0.01	0.01	0.01
Rikenella	<0.01	<0.01	0.01	<0.01	0.01	0.01	<0.01	<0.01
Pseudomonas	<0.01	<0.01	<0.01	0.01	0.01	0.02	<0.01	0.01
Citrobacter	<0.01	<0.01	<0.01	<0.01	<0.01	<0.01	0.03	0.06
UCG-004	<0.01	<0.01	0.01	0.02	<0.01	<0.01	0.01	0.02
Atopostipes	<0.01	<0.01	<0.01	<0.01	<0.01	<0.01	0.02	0.02
Brachybacterium	<0.01	<0.01	<0.01	<0.01	<0.01	<0.01	0.02	0.05
Muribaculum	<0.01	<0.01	<0.01	<0.01	0.03	0.03	<0.01	<0.01
Globicatella	<0.01	<0.01	<0.01	<0.01	<0.01	<0.01	0.02	0.03
Holdemania	<0.01	<0.01	<0.01	0.01	0.01	0.01	<0.01	<0.01
Mogibacterium	<0.01	<0.01	<0.01	<0.01	<0.01	<0.01	0.02	0.01
Oceanobacillus	<0.01	<0.01	<0.01	<0.01	<0.01	<0.01	0.01	0.01
Paraprevotella	<0.01	<0.01	<0.01	<0.01	0.03	0.04	<0.01	<0.01
Elusimicrobium	<0.01	<0.01	<0.01	<0.01	0.01	0.02	<0.01	<0.01
Enteractinococcus	<0.01	<0.01	<0.01	<0.01	<0.01	<0.01	0.01	0.01
Proteus	<0.01	0.01	<0.01	<0.01	<0.01	<0.01	0.01	0.01
Brevibacterium	<0.01	<0.01	<0.01	<0.01	<0.01	<0.01	0.01	0.01
Lautropia	<0.01	<0.01	<0.01	<0.01	<0.01	<0.01	0.02	0.03
Actinomyces	<0.01	<0.01	<0.01	<0.01	<0.01	<0.01	0.01	0.03
Clostridium_sensu_stricto_13	<0.01	<0.01	<0.01	<0.01	<0.01	<0.01	0.01	0.02
Clostridium_sensu_stricto_11	<0.01	<0.01	<0.01	<0.01	<0.01	<0.01	0.01	0.02
Morganella	<0.01	<0.01	<0.01	<0.01	<0.01	<0.01	0.01	0.01

Supplementary Table S5. The relative abundance of major genera at different ages (average relative abundance > 0.01 %). mean[a], average relative abundance; SD[b], standard deviation.
